# Towards Sustainable Composite Structures: Experimental Characterisation and Damage Modelling of Unidirectional Flax Fibre-Reinforced Polymers

**DOI:** 10.3390/polym17222985

**Published:** 2025-11-10

**Authors:** Martin Stejskal, Frantisek Sedlacek, Ondrej Spacek, Nikola Bednarova, Jan Krystek

**Affiliations:** 1 Faculty of Mechanical Engineering, University of West Bohemia, Univerzitni 2732/8, 301 00 Pilsen, Czech Republic; fsedlace@fst.zcu.cz (F.S.); spaceko@fst.zcu.cz (O.S.); bednaron@fst.zcu.cz (N.B.); 2 Faculty of Applied Sciences, University of West Bohemia, Univerzitni 2732/8, 301 00 Pilsen, Czech Republic; krystek@kme.zcu.cz

**Keywords:** flax fibre-reinforced polymers, experimental testing, mechanical properties, intralaminar damage, virtual prototyping, OHT, FEA, CDM

## Abstract

The increasing demand for sustainable engineering solutions has driven extensive research into natural fibre-reinforced composites (NFCs), notably flax fibre-reinforced polymers (FFRPs), which offer promising eco-friendly alternatives to synthetic composites. This study presents a comprehensive application of continuum damage mechanics (CDM) methodology to unidirectional (UD) FFRPs, addressing critical gaps in predictive modelling of progressive intralaminar damage for sustainable structural applications. A systematic experimental characterisation protocol was developed to identify material parameters that account for the inherent variability and complex nonlinear behaviour of natural fibres. The CDM model was calibrated using comprehensive quasi-static testing on multiple laminate configurations and validated through finite element analysis (FEA) in Siemens Simcenter Samcef. The model accurately captures the nonlinear behaviour and damage mechanisms of notched flax–epoxy laminates, achieving prediction accuracies of 97.61% and 88.98% for the force-displacement response in open-hole tensile (OHT) validation tests. Integrating experimental characterisation with FEA enables robust virtual prototyping of sustainable composite structures, supporting design optimisation and lifecycle assessment. This research establishes validated damage modelling methodologies for eco-friendly NFC, facilitating accelerated adoption in automotive, marine, and construction sectors.

## 1. Introduction

Growing environmental consciousness and legislative pressure have positioned natural fibre-reinforced composites (NFCs) as key materials for sustainable engineering applications [1,2,3,4]. Natural fibres, such as flax, jute and hemp, offer distinct advantages over synthetic alternatives, including biodegradability, lower density, reduced carbon footprint during production, and competitive mechanical properties. Despite the benefits, NFCs present unique challenges related to their inherent variability, moisture sensitivity, and complex damage mechanisms [1,2,3,4,5].

Recent comprehensive reviews have confirmed the widespread adoption of NFCs across multiple industries, particularly in construction and aerospace [1,3], automotive [4,6], and marine [7] sectors. Flax fibre-reinforced composites (FFRPs) have applications in automotive interior components, structural components, acoustic panels, and even load-bearing elements due to their high specific stiffness and damping properties [4]. Life cycle assessments (LCA) consistently demonstrate that FFRP production results in significantly lower environmental impact compared to conventional glass fibre composites (GFRPs), with up to 50% reduction in CO_2_ emissions [8]. Market adoption has accelerated with major manufacturers integrating NFCs into commercial products, driven by environmental regulations and consumer demand for sustainable alternatives [3,4].

Experimental characterisation of FFRPs has extensively documented their mechanical, thermal, fatigue, and impact properties under various loading conditions [9,10,11,12,13,14,15,16]. Studies have shown that flax fibres exhibit specific properties comparable to E-glass in certain applications, making them viable candidates for structural use [17]. Research has focused on optimising fibre–matrix adhesion through various surface treatments, including alkali treatment, silane coupling, and plasma modification, to enhance mechanical performance and reduce moisture sensitivity [18,19,20,21]. Dynamic mechanical analysis has revealed that FFRPs possess excellent vibration-damping characteristics, making them particularly suitable for noise and vibration control applications [4,17,22].

Progressive damage modelling represents a critical research gap in the development of NFCs. Synthetic fibre composites benefit from well-established failure criteria [23,24,25,26], such as Tsai-Wu [27], Hashin [28], Puck [29], and LaRC [30,31,32] models. Recent advances [33,34,35,36,37,38,39] have demonstrated the successful implementation of the continuum damage mechanics (CDM) theory established by Kachanov [40], with the Ladevèze–LeDantec [41] mesoscale damage theory showing particular promise for future adaptation to NFCs.

Multiscale finite element analysis (FEA) approaches have been developed to capture the progressive damage of FFRPs, ranging from micro-scale fibre–matrix debonding to macro-scale laminate failure [15,16,42,43,44,45,46]. Monte Carlo simulations have accounted for the natural variability in fibre properties and geometric irregularities [47]. Machine learning approaches are increasingly integrated with experimental data to develop predictive models that accommodate the inherent scatter in natural fibre properties [48,49]. These advanced modelling techniques have enabled virtual prototyping capabilities that reduce experimental requirements and optimise composite design.

Experimental validation remains crucial for damage model development, with robust parameter identification procedures [50,51,52] and standardised testing protocols, including ASTM D3039 [53], D7264 [54], and D3410 [55], being adapted for NFCs [56,57]. Low-velocity impact testing has revealed unique damage mechanisms in FFRPs, including hierarchical fibre failure and moisture-dependent behaviour [12,58,59]. Quasi-static testing under various loading conditions has provided insights into the complex, nonlinear behaviour of FFRPs, including plasticity and damage-coupling effects [11,13,60]. In situ testing and high-speed imaging have directly observed damage evolution mechanisms at multiple length scales [61].

The field continues to debate several controversial aspects regarding NFC modelling. The primary controversy centres on the reliability of predictive models for NFCs [47,62], given their high variability [2,3,4,5]. Some researchers advocate for probabilistic approaches [47,62], while others focus on deterministic models with safety factors [48,63]. Disagreement exists regarding the optimal failure criteria for capturing the complex damage mechanisms in natural fibres, notably the transition from elastic to plastic behaviour [9,10,11,16]. The influence of moisture content on damage evolution remains poorly understood, with conflicting reports on its effects on composite durability [2,12]. Hybridisation strategies combining natural and synthetic fibres present another area of ongoing research [47,64], with studies showing both positive [17] and negative [65] synergistic effects, depending on the specific combination and manufacturing methods.

This manuscript addresses a critical knowledge gap in sustainable composites by developing and validating advanced damage modelling methodologies tailored explicitly for FFRP laminates used in eco-friendly structural applications. While numerous studies have focused on basic characterisation and manufacturing aspects of NFCs, few have systematically integrated experimental findings with CDM-based progressive damage models for design applications [16]. Previous NFC modelling efforts have been limited by (1) reliance on simplified failure criteria (e.g., maximum stress, Tsai-Wu) that cannot capture progressive damage evolution and nonlinear material behaviour, (2) lack of comprehensive parameter identification procedures adapted to the high variability and complex damage mechanisms of natural fibres, and (3) insufficient validation for stress-concentration scenarios relevant to practical structural applications. Unlike phenomenological failure criteria, which predict only ultimate failure loads, CDM models capture the complete nonlinear stress–strain response, including damage initiation, evolution, and ultimate failure, while accounting for coupling between damage and plasticity mechanisms. This capability is essential for virtual prototyping, design optimisation, and reliable performance prediction of sustainable composite structures. The present work bridges fundamental damage mechanics theory with practical engineering requirements for NFC design by establishing validated procedures for model calibration and demonstrating predictive capabilities on notched laminates.

In this paper, the PLYUNI3 model is calibrated using the commercial solver Siemens Simcenter Samcef 2412 [66] for FLAXPREG T-UD 100, which is a FFRP with 100 $g/m^{2}$ pre-impregnated unidirectional (UD) reinforcement and epoxy resin in a 1:1 weight ratio. The work involves comprehensive experimental testing, including monotonic and cyclic, tensile and compressive quasi-static loading of various laminate configurations (0°, 90°, ±45°, ±22.5°), parameter identification procedures following established protocols, and validation through open-hole tensile tests (OHT).

The advanced CDM model accurately captures the nonlinear progressive damage behaviour of FFRPs, including matrix microcracking, fibre–matrix debonding, and fibre failure mechanisms. Comprehensive experimental validation using standardised test protocols demonstrates the modelling approach’s capabilities and limitations for various loading scenarios. Integrating experimental material characterisation with FEA provides a robust framework for virtual prototyping of eco-friendly composite structures. These findings establish a foundation for future research on optimisation strategies, environmental effect modelling, and lifecycle prediction, which are essential for the broader adoption of sustainable composites in structural applications.

## 2. Progressive Damage Modelling of Elementary Ply

This study focuses on the intralaminar mesoscale, plane-stress damage model for a homogeneous elementary ply of a transversely isotropic UD composite proposed by Ladevèze and LeDantec [41]. This CDM-based model accounts for matrix microcracking, fibre matrix debonding, and brittle fibre fracture. The fibre fracture is assumed to be instantaneous, whereas linear damage laws describe the initiation and evolution of other failure mechanisms. The progressive damage is characterised by internal stiffness degradation coupled with plasticity, capturing the nonlinear behaviour due to internal friction. In [41], the strain energy in the elastic regime is described as

(1) $$E_{D}=\frac{1}{2}\left[\frac{\sigma_{11}^{2}}{E_{1}^{0}}-2\frac{\nu_{12}^{0}}{E_{1}^{0}}\sigma_{11}\sigma_{22}+\frac{{\left\langle \sigma_{22}\right\rangle }_{+}^{2}}{E_{2}^{0}\left(1-d_{22}\right)}+\frac{{\left\langle \sigma_{22}\right\rangle }_{-}^{2}}{E_{2}^{0}}+\frac{\tau_{12}^{2}}{G_{12}^{0}\left(1-d_{12}\right)}\right],$$

where σ and $\tau_{12}$ are normal and shear stresses in the lamina principal direction, $E^{0}$, $G_{12}^{0}$, and $\nu_{12}^{0}$ are undamaged elastic properties, and $d_{22}$ and $d_{12}$ are shear and transverse scalar damage variables, respectively. Macaulay brackets indicate the non-smooth ramp function, separating the transverse strain energy into positive and negative components:

(2) $${\left\langle \sigma \right\rangle }_{+}=\sigma \;\mathrm{when}\;\sigma \geq 0;\;\mathrm{otherwise}\;{\left\langle \sigma \right\rangle }_{+}=0,\;{\left\langle \sigma \right\rangle }_{-}=\sigma \;\mathrm{when}\;\sigma \leq 0;\;\mathrm{otherwise}\;{\left\langle \sigma \right\rangle }_{-}=0.$$

It is assumed that matrix microcracks close in compression; thus, the evolution of transverse damage is defined solely in tension. Shear damage in the form of fibre matrix debonding leads to material degradation regardless of the load type. The individual components of elastic deformation are derived from Hooke’s law:

(3) $$\varepsilon^{e}=S\sigma =\frac{\partial E_{D}}{\partial \sigma }↔\left\{\begin{array}{c} \varepsilon_{11}^{e}=\frac{\sigma_{11}}{E_{1}^{0}}-\frac{\nu_{12}^{0}}{E_{1}^{0}}\sigma_{22},\\ \varepsilon_{22}^{e}=\frac{{\left\langle \sigma_{22}\right\rangle }_{+}}{E_{2}^{0}\left(1-d_{22}\right)}+\frac{{\left\langle \sigma_{22}\right\rangle }_{-}}{E_{2}^{0}}-\frac{\nu_{12}^{0}}{E_{1}^{0}}\sigma_{11}\\ \gamma_{12}^{e}=\frac{\tau_{12}}{G_{12}^{0}\left(1-d_{12}\right)}. \end{array}\right.,$$

The damage evolution is governed by thermodynamic forces $Y_{22}$ and $Y_{12}$, which are analogous to energy release rates for crack growth. They are defined as follows:

(4) $$Y_{22}=\frac{\partial E_{D}}{\partial d_{22}}=\frac{{\left\langle \sigma_{22}\right\rangle }_{+}^{2}}{2E_{2}^{0}(1-d_{22}{)}^{2}},$$

(5) $$Y_{12}=\frac{\partial E_{D}}{\partial d_{12}}=\frac{\tau_{12}^{2}}{2G_{12}^{0}(1-d_{12}{)}^{2}}.$$

Based on experimental observations, Ladevèze and LeDantec [41] described the evolution of failure mechanisms over the time t as

(6) $$\underline{Y_{22}}\left(t\right)=\underset{\tau \leq t}{\sup}\sqrt{Y_{22}\left(\tau \right)},$$

(7) $$\underline{Y}\left(t\right)=\underset{\tau \leq t}{\sup}\sqrt{Y_{12}\left(\tau \right)+b_{2}Y_{22}\left(\tau \right)}.$$

The time evolution of the damage variables is assumed to be linear:

(8) $$d_{22}\left(t\right)=\left\{\begin{array}{ll} \frac{{\left\langle \underline{Y}\left(t\right)-Y_{22\;0}\right\rangle }_{+}}{Y_{22\;c}} & when\;d_{22}<1\;and\;\underline{Y_{2}}\left(t\right)\leq Y_{22s},\\ 1 & \mathrm{otherwise}, \end{array}\right.$$

(9) $$d_{12}\left(t\right)=\left\{\begin{array}{ll} \frac{{\left\langle \underline{Y}\left(t\right)-Y_{12\;0}\right\rangle }_{+}}{Y_{12\;c}} & {when\;d}_{12}<1\;and\;\underline{Y_{2}}\left(t\right)\leq Y_{22s},\\ 1 & \mathrm{otherwise}, \end{array}\right.$$

where $Y_{22\;0}$ and $Y_{12\;0}$ are the initiation thresholds, $Y_{22c}$ and $Y_{12c}$ are the critical values for complete damage, and $Y_{22\;s}$ is the ultimate threshold for brittle fracture at the fiber–matrix interface in transverse tension. Damage in the elementary ply is assumed to be continual and irreversible, and the internal stiffness degradation is reflected in the decrease of the corresponding moduli:

(10) $$E_{2}\left(t\right)=E_{2}^{0}\left(1-d_{22}\right),$$

(11) $$G_{12}\left(t\right)=G_{12}^{0}\left(1-d_{12}\right).$$

To model permanent plastic deformation resulting from internal friction, Ladevèze and LeDantec [41] introduced the following plasticity law consisting of an elastic domain function f and an isotropic hardening law $R\left(p\right)$:

(12) $$f=\sqrt{{\tilde{\tau }}_{12}^{2}+a^{2}{\tilde{\sigma }}_{22}^{2}}-R\left(p\right)-R_{0},$$

(13) $$R\left(p\right)=Kp^{\gamma },$$

where a is the shear-transverse coupling constant, p is the accumulated plastic strain, $R_{0}$ is the yield stress, and K and γ are the power-law hardening parameters. The plasticity in this model is based on the effective values of stress and strain:

(14) $$\tilde{\sigma }=\left[\begin{array}{c} \sigma_{11}\\ \frac{{\left\langle \sigma_{22}\right\rangle }_{+}}{1-d_{22}}+{\left\langle \sigma_{22}\right\rangle }_{-}\\ \frac{\tau_{12}}{1-d_{12}} \end{array}\right],$$

(15) $${\dot{\tilde{\varepsilon }}}^{p}=\left[\begin{array}{c} {\dot{\varepsilon }}_{11}^{p}\\ {\dot{\varepsilon }}_{22}^{p}\left[\frac{{\left\langle \sigma_{22}\right\rangle }_{+}}{\sigma_{22}}\left(1-d_{22}\right)+\frac{{\left\langle \sigma_{22}\right\rangle }_{-}}{\sigma_{22}}\right]\\ {\dot{\gamma }}_{12}^{p}\left(1-d_{12}\right) \end{array}\right],$$

which are linked to the plastic dissipation rate $\phi_{p}$:

(16) $$\phi_{p}=Tr\left[\tilde{\sigma }{\dot{\tilde{\varepsilon }}}^{p}\right]=Tr\left[\sigma {\dot{\varepsilon }}^{p}\right].$$

The model assumes no plastic yielding in the fibre direction and neglects the influence of stress in the fibre direction on plastic development. Therefore, the yield conditions can be written as

(17) $${\dot{\tilde{\varepsilon }}}^{p}=\dot{p}\frac{\partial f}{\partial \tilde{\sigma }}↔\left\{\begin{array}{c} {\dot{\tilde{\varepsilon }}}_{11}^{p}=0,\\ {\dot{\tilde{\varepsilon }}}_{22}^{p}=a^{2}\dot{p}\frac{{\tilde{\sigma }}_{22}}{R+R_{0}},\\ {\dot{\tilde{\gamma }}}_{12}^{p}=\dot{p}\frac{{\tilde{\tau }}_{12}}{R+R_{0}}, \end{array}\right.$$

where $\dot{p}$ is the rate of the plastic strain accumulation defined as

(18) $$\dot{p}=\left\{\begin{array}{ll} \frac{{\tilde{\tau }}_{12}{\dot{\tilde{\tau }}}_{12}+a^{2}{\tilde{\sigma }}_{22}{\dot{\tilde{\sigma }}}_{22}}{\left(R+R_{0}\right)\frac{\partial R}{\partial p}}=\sqrt{{{\dot{\tilde{\gamma }}}_{12}^{p}}^{2}+a^{2}{{\dot{\tilde{\varepsilon }}}_{22}^{p}}^{2}} & when\;f=\dot{f}=0;\\ 0 & \mathrm{otherwise}. \end{array}\right.$$

During experimental testing, Ladevèze and LeDantec [41] observed differences in the linear and nonlinear behaviour of composites subjected to tensile and compressive loading along the fibre direction. This phenomenon is modelled by introducing a parameter $\xi^{-}$ for nonlinear stiffness loss in compressive loading:

(19) $$E_{1}=E_{1}^{0}\left(1+\xi^{-}{\langle \sigma_{11}\rangle }_{-}\right).$$

The intralaminar model proposed by Ladevèze and LeDantec [41] was initially used to predict the failure of carbon fibre-reinforced composites (CFRP) IM6/914 and T300/914. Since then, Allix and Ladevèze [67] addressed the absence of an interlaminar degradation mechanism. Lubineau and Ladevèze [68] described the transverse cracking of UD composites as a function of crack density to model the through-thickness propagation of diffuse damage based on micro-mechanical considerations. Hochard et al. [69] modified the model for woven-fabric (WF) composites.

The intra- and interlaminar models were implemented and verified in the commercial finite element (FE) solver Samcef by Bruyneel et al. [35,36,70] for both UD and WF composites. In the current version of Siemens Simcenter Samcef [66], there are multiple versions of the CDM intra-laminar models—PLYUNI, which extends the original formulation [41] by including a damage evolution law in the fibre direction, and PLYENH, which uses the enhanced version [68] by describing the transverse microcracking as a function of crack density. These models natively support through-thickness damage propagation, damage rate laws to ease convergence and reduce computational complexity, and damage localisation to allow intra- and inter-model coupling, to model the ply–interface interaction. However, they are also implemented in other commercial FE codes, such as Abaqus, LS-Dyna, and PAM-Crash.

Despite the advancements, extensive experimental testing is required to obtain the necessary material properties. Therefore, robust parameter identification procedures have been proposed by O’Higgins et al. [50], Daghia and Ladevèze [51], and Malgioglio et al. [52] to enhance repeatability and scalability. For comprehensive material characterisation, it is recommended to examine ${\left[0{}^{\circ}\right]}_{8}$, ${\left[90{}^{\circ}\right]}_{16}$ and ${\left[\pm 45{}^{\circ}\right]}_{2s}$ laminates in tension, ${\left[0{}^{\circ}\right]}_{8}$ and ${\left[90{}^{\circ}\right]}_{16}$ laminates in compression, as well as ${\left[\pm 45{}^{\circ}\right]}_{2s}$ and ${\left[\pm 67.5{}^{\circ}\right]}_{2s}$ laminates under cyclic loading, while prior knowledge of elastic and strength properties may allow the use of cross-ply laminates to reduce the total number of experiments [41,50]. Abisset et al. [33] demonstrated that open-hole tensile testing can be used to validate identified parameters.

Recently, Rajaneesh et al. [38] introduced a damage-rate-bound regularisation and demonstrated accurate strength prediction for scaled open-hole laminates. In further work, Rajaneesh et al. [39] presented a physically based modification for failure analysis of WF composites. Cherniaev et al. [37] demonstrated the model’s prediction capabilities for the axial crushing response of flat and C-channel CFRP composites under dynamic loading.

The benefits of the CDM approach over simplified failure criteria (e.g., maximum stress, Tsai-Wu) for structural design include the following: (1) prediction of complete nonlinear force-displacement response rather than only ultimate failure loads, enabling the assessment of structural stiffness degradation and damage tolerance, (2) capture of progressive damage evolution and interaction between different failure mechanisms (matrix cracking, fibre–matrix debonding, fibre failure), (3) prediction of spatial damage distributions and failure mode transitions, which are essential for design optimisation and damage-tolerant structural concepts, and (4) applicability to complex loading scenarios and geometric stress concentrations where simple failure criteria are known to be inaccurate. While the additional experimental effort for CDM calibration is significant, the methodology enables advanced virtual prototyping capabilities that reduce the number of expensive structural-level validation tests required during the design development process.

While the Ladevèze–LeDantec model was originally developed for synthetic fibre composites with linear elastic fibres and distinct fibre–matrix interfaces, its application to natural fibre composites requires careful consideration of fundamental assumptions. Flax fibres exhibit viscoelastic behaviour, nonlinear stress–strain response, and complex hierarchical microstructure (cell wall layers, lumen, microfibrils) that differ significantly from synthetic fibres. However, the CDM approach operates at the mesoscale, treating the elementary ply as a homogenised continuum rather than explicitly modelling individual fibre and matrix constituents. At this scale, the effective nonlinear behaviour arising from fibre viscoelasticity, progressive interface failure, and microstructural evolution is captured through the calibrated damage and plasticity parameters rather than requiring modification of the fundamental model equations. The coupling between damage and plasticity, along with the isotropic hardening law, provides sufficient flexibility to represent the complex inelastic behaviour of FFRPs. The modelling of rate-dependent effects and long-term creep behaviour would require additional constitutive features (e.g., viscoplasticity) beyond the current model capabilities.

## 3. Materials and Methods

**Figure 1:**
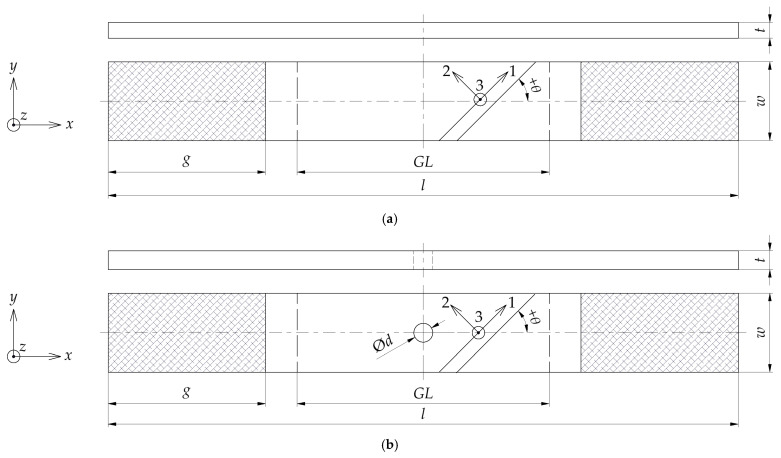
General test specimen geometry for experimental testing (x, y, z–test CS; 1, 2, 3–laminate CS): (**a**) specimen geometry for tensile and compressive testing; (**b**) specimen geometry for OHT.

The general test specimen geometry for experimental testing is depicted in Figure 1. Detailed information regarding the experimental test matrix, including specimen dimensions, stacking sequences, and instrumentation used, is provided in Table 1. The test matrix was designed to determine the required material properties for damage modelling of FFRPs via monotonous and cyclic quasi-static tensile and compressive testing, with validation on OHT. Where feasible, standardised procedures were adhered to—ASTM D3039 [53], D3518 [71] and D5766 [72] for tensile loading; ASTM D3410 [55] for compressive loading.

All test specimens were manufactured at the University of West Bohemia. First, composite panels with the required lay-ups were hand-laminated from FLAXPREG T-UD 100 prepreg sheets and cured in an autoclave for 1 h at 120 °C at a pressure of 3 bar, following the manufacturer’s recommendations and ASTM D5687 [73], resulting in a nominal fibre volume fraction of approximately 48 ± 2%. The cured laminate density was measured as 1.35 ± 0.03 g/cm^3^. These panels were then sectioned into individual specimens using an industrial waterjet cutting machine WJ2830-2Z-Cobra-PJ60° (manufactured by PTV in Hostivice, Czech Republic); plywood panels were used to prevent unwanted deformation. Steel tabs were glued onto the specimens for compressive tests using a two-component epoxy adhesive LOCTITE® EA 9464 (manufactured by Henkel in Duesseldorf, Germany), and centred holes were milled into the specimens used for OHT validation tests. All specimens were stored under laboratory conditions at 20 ± 2 °C and 50 ± 5% relative humidity. The natural moisture content in the prepreg material and cured specimens was not controlled or measured, representing typical handling conditions for these materials in practical applications.

To verify the accuracy of the manufacturing process, the dimensions of test specimens were measured using laboratory-grade equipment. These measurements revealed a negligible geometric deviation of less than ± 0.1 mm in width and length. The thickness varied from 1.244 mm to 1.385 mm, with a mean value of 1.322 ± 0.076 mm (CV of 2.9%). The OHT specimens were also manufactured with precision—the diameters of the holes ranged from 3.91 mm to 3.99 mm, with a mean value of 3.95 ± 0.04 mm (CV of 0.56%), while the tolerance for the centre position of the holes was ± 0.16 mm. The gauge length was 50 ± 0.1 mm or 75 ± 0.1 mm for specimens instrumented with AE.

In total, 56 specimens were fabricated to repeat each test configuration three to eight times. Although the sample size may appear limited for FFRPs, the test matrix was designed based on standard practice for calibrating progressive damage models, as established for synthetic composites. The statistical analysis (mean ± 2 × SD and CV) provided a quantitative assessment of specimen-to-specimen experimental variability based on the tested sample size and is consistent throughout this study. The SD and CV characterise the material property scatter from natural fibre heterogeneity, manufacturing variations, and experimental measurement uncertainty. The fitted parameter values from linear regression (e.g., damage and plasticity parameters) represent the best-fit values from individual specimen curves, with the reported statistics quantifying the variation in these fitted parameters between specimens. This statistical reporting approach is consistent with standard practice for characterising composite materials and provides essential information for reliability assessment and safety factor determination in engineering applications.

## 4. Experimental Testing

**Figure 2:**
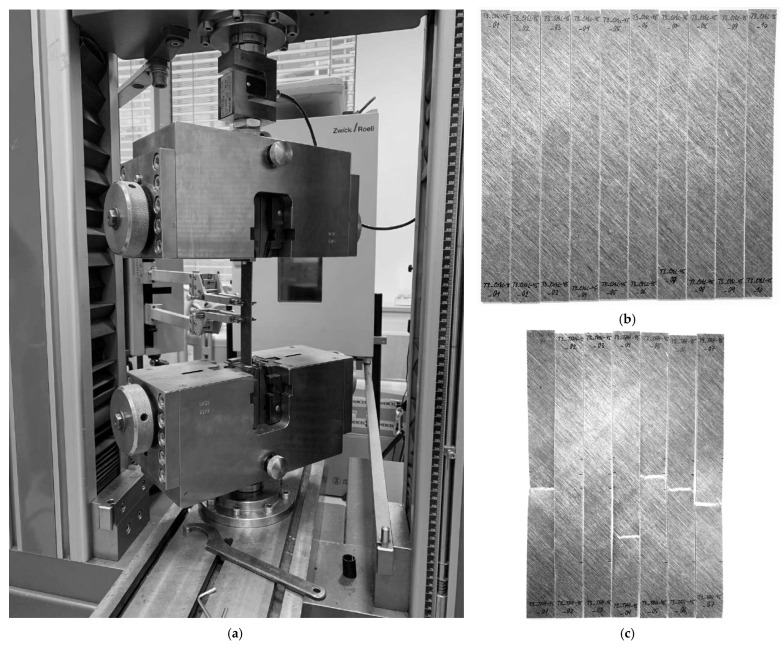
Experimental testing of composite laminate test specimens: (**a**) experimental testing setup; (**b**) specimens before experimental testing; (**c**) specimens after experimental testing.

The mechanical testing of the FFRP specimens was conducted at the University of West Bohemia using a universal testing machine Zwick/Roell Z050 (manufactured by Zwick Roell Group in Ulm, Germany) with a maximum tensile force of 50 kN. The specimens were loaded to failure at a displacement rate of 2 mm/min for tensile and compressive tests, and 1.27 mm/min for open-hole tensile tests. The cyclic tests consisted of 5 to 8 cycles of controlled loading and unloading, during which the load amplitude was increased for each cycle up to failure. The load increment was set based on the results of preceding monotonous tests. The unloading rate was set to be 2 to 3 times faster than the loading rate to reduce the overall testing time. All mechanical testing was performed under controlled laboratory conditions at 20 ± 2 °C and 50 ± 5% relative humidity, with no additional moisture conditioning, using calibrated equipment. The experimental setup and tested specimens are depicted in Figure 2.

The damage initiation and progression were monitored via continuous two-sided imaging during testing. Visual inspection was performed on all specimens before and after testing to identify failure modes and damage patterns. For the CDM model parameter identification, damage initiation and progression were indirectly characterised by stress–strain behaviour analysis during cyclic loading. Specifically, damage evolution was quantified from the reduction in reloading modulus compared to the initial elastic modulus. At the same time, permanent plastic deformation was measured from strain offsets at 50 N pre-load during unloading cycles. This approach follows established procedures for CDM parameter identification and does not require direct observation of micro-scale damage mechanisms during testing.

## 5. Identification of Material Properties

The identification of material properties for CDM model calibration requires systematic experimental testing combined with analytical post-processing of stress–strain data. This section outlines the procedures for extracting elastic and strength properties, as well as damage, plasticity, and coupling parameters, from the quasi-static testing of various laminate configurations. The identification process follows established protocols. Section 5.1 presents the determination of basic elastic and strength parameters from monotonic testing, while Section 5.2 describes the more complex procedure for identifying damage and plasticity parameters from cyclic loading tests. The complete set of 28 model parameters (Table 2, Table 3, Table 4, Table 5, Table 6, Table 7, Table 8 and Table 9) provides a comprehensive material characterisation, enabling virtual prototyping of FFRP structures.

### 5.1. Elasticity and Strength Parameters

The elasticity and strength parameters of FFRPs were identified from experimental stress–strain curves following the recommendations in ASTM standards [53,55,71].

**Figure 3:**
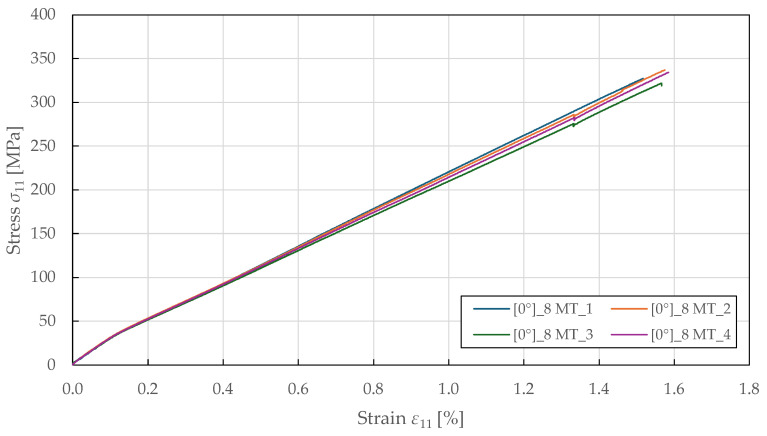
Experimental fibre-direction stress–strain response of FFRPs to MT loading.

**Figure 4:**
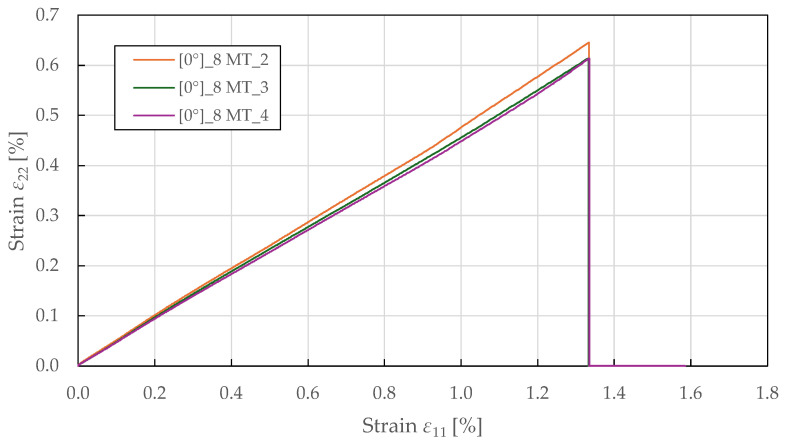
Experimental fibre-direction transverse-longitudinal strain response of FFRPs to MT loading.

The tensile fibre-direction properties were determined using ${\left[0{}^{\circ}\right]}_{8}$ specimens tested in accordance with ASTM D3039 [53]. The resulting stress–strain curves in Figure 3 demonstrate a bi-linear response up to failure, yielding around a stress of 35 ± 2 MPa and a strain of 0.12 ± 0.01%, beyond which the response becomes nonlinear with a reduced tangent modulus. This bi-linear behaviour differs from that of carbon fibre composites, which typically exhibit a linear response to failure, and reflects the progressive accumulation of damage in the form of microcracking at the fibre–matrix interface and internal fibre damage. The yield point represents the onset of plastic deformation, beyond which permanent damage accumulates. The specimen-to-specimen variability in the post-yield behaviour is characteristic of natural fibre composites and reflects heterogeneity in fibre properties, waviness, and manufacturing quality. The relationship between transverse and longitudinal strain is predominantly linear, as shown in Figure 4. The identified Poisson’s ratio ($\nu_{12}=0.48\pm 0.05$) indicates strong coupling between longitudinal extension and transverse contraction. This high value of Poisson’s ratio (compared to typical values of 0.25–0.35 for synthetic fibre composites) may reflect the complex deformation mechanisms of flax fibres, including microstructural rearrangement and matrix plastic flow. The biaxial extensometer was unclipped at a longitudinal and transverse strain of 1.32 ± 0.05% and 0.57 ± 0.03%, respectively, to avoid damaging it, well before ultimate failure at 1.56 ± 0.06%. This confirms that the linear strain relationship is maintained throughout the majority of the loading history. The identified tensile fibre-direction material properties for the CDM model are summarised in Table 2.

**Figure 5:**
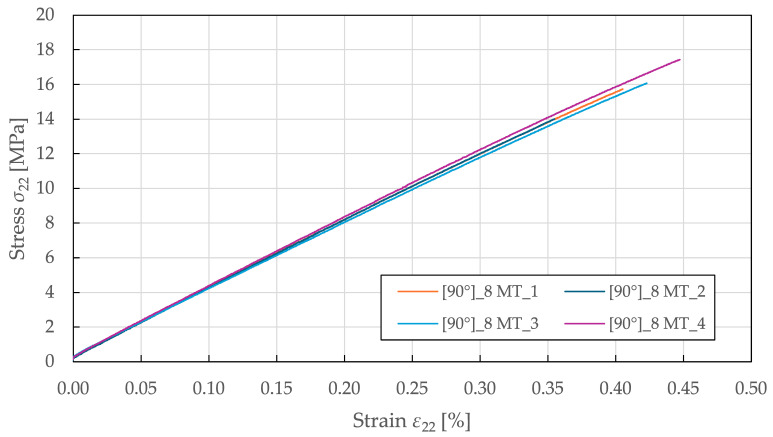
Experimental transverse direction stress–strain response of FFRPs to MT loading.

The tensile transverse direction properties were characterised using ${\left[90{}^{\circ}\right]}_{8}$ specimens tested per ASTM D3039 [53]. As shown in Figure 5, the obtained stress–strain curves exhibit a predominantly linear response up to failure. Starting at a stress and strain of 5 ± 1 MPa and 0.13 ± 0.02%, the responses deviate from the initial linearity, resulting in a slight loss of stiffness. The identified tensile transverse direction material properties for the CDM model are summarised in Table 3.

**Figure 6:**
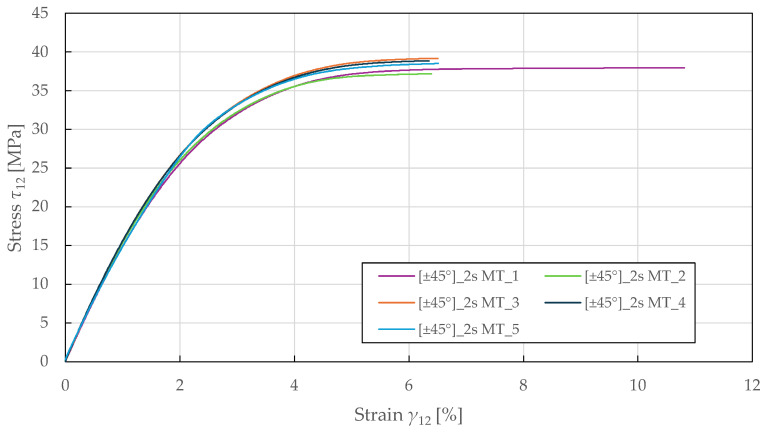
Experimental in-plane shear stress–strain response of FFRPs.

The in-plane shear properties were determined from tensile testing of ${\left[\pm 45{}^{\circ}\right]}_{2s}$ specimens according to ASTM D3518 [71]. The resulting response in Figure 6 demonstrates a nonlinear behaviour up to failure, with a yield point at a stress and strain of 24.5 ± 0.5 MPa and 1.80 ± 0.05%. The identified in-plane shear material properties for the CDM model are summarised in Table 4.

**Figure 7:**
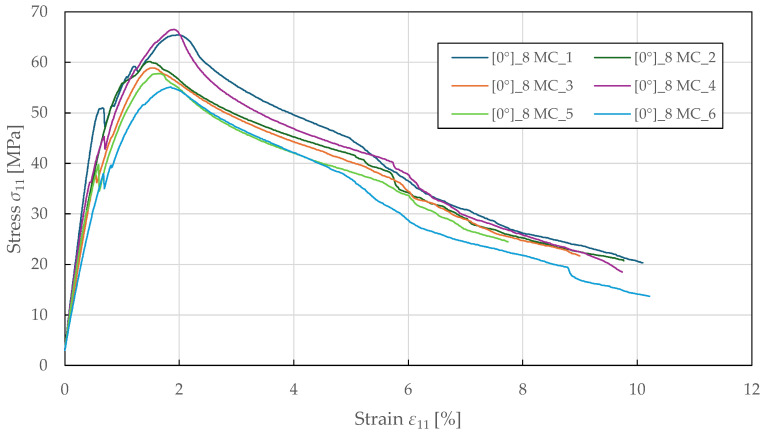
Experimental fibre-direction stress–strain response of FFRPs to MC loading.

The compressive fibre-direction properties were determined from ${\left[0{}^{\circ}\right]}_{8}$ specimens tested in accordance with ASTM D3410 [55]. The experimental stress–strain curves shown in Figure 7 exhibit a nonlinear response up to failure. Initially, the curves show a linear behaviour, followed by a sudden yield point at a stress and strain of 42.5 ± 8.0 MPa and 0.64 ± 0.05%. This likely indicates the onset of matrix microcracking or early fibre/matrix debonding. Beyond the peak stress region, the curves exhibit a softening behaviour characterised by a gradual decline in load-bearing capacity, reflecting loss of stability and bending of the specimens, which occurred consistently across all samples. The failure was observed at an ultimate strain of 9.5 ± 0.6%, far exceeding the strain at maximum stress. The identified compressive fibre-direction material properties for the CDM model are summarised in Table 5.

**Figure 8:**
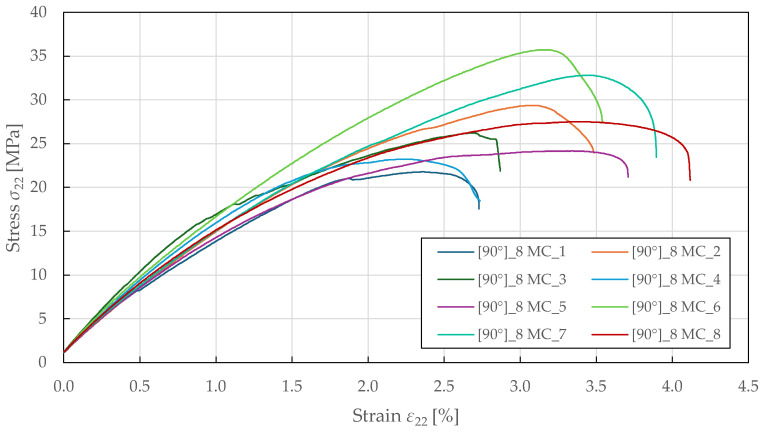
Experimental transverse direction stress–strain response of FFRPs to MC loading.

The tensile transverse direction properties were characterised using ${\left[90{}^{\circ}\right]}_{8}$ specimens tested per ASTM D3410 [55]. As shown in Figure 8, the obtained response exhibits a nonlinear behaviour up to failure, with yielding occurring at a stress and strain of approximately 15.0 ± 1.5 MPa and 1.00 ± 0.08%. Post-peak, the curves show a sudden decrease in load-bearing capacity, indicating stable crushing, with a notable specimen-to-specimen variability in strength and ductility. The identified compressive transverse direction material properties for the CDM model are summarised in Table 6.

The identified material parameters of FFRPs reveal characteristic damage anisotropy patterns that differ from those of typical synthetic fibre composites. The ratio of longitudinal to transverse tensile modulus ($E_{1}$/$E_{2}$ = 7.3) is lower than typical CFRPs (typically 10–15) but higher than GFRPs (typically 3–5), reflecting the moderate stiffness anisotropy of flax fibres. The ratio of tensile to compressive strength in the fibre direction ($X_{T}$/$X_{C}$ = 5.4) is significantly higher than that of CFRP (typically 1.5–2.0), indicating the relatively poor compressive performance of natural fibres due to kinking and microbuckling facilitated by their cellular structure. The transverse tensile-to-compressive strength ratio ($Y_{T}$/$Y_{C}$ = 0.57) also differs from synthetic composites, where transverse compression strength typically exceeds tension strength due to crack closure effects. In FFRPs, the porous structure and lower fibre–matrix interfacial strength result in progressive compressive damage under transverse compression, which the current model formulation does not adequately capture. The relatively high shear-to-transverse modulus ratio ($G_{12}$/$E_{2}$ = 0.4) compared to CFRP (typically 0.2–0.3) reflects the lower transverse stiffness and different deformation mechanisms of natural fibres. These anisotropy characteristics have important implications for design, as composite structures using FFRPs must be configured to minimise compressive loading perpendicular to the fibre direction.

### 5.2. Damage and Plasticity Parameters

The damage, plasticity, and coupling parameters of FFRPs were identified following the procedure outlined in [41] from cyclic tensile testing of specimens with ${\left[0{}^{\circ}\right]}_{8}$, ${\left[\pm 45{}^{\circ}\right]}_{2s},$ and ${\left[\pm 22.5{}^{\circ}\right]}_{2s}$ lay-ups. In the CDM model, progressive damage is characterised as a loss of moduli, which reflects material stiffness, and is revealed from hysteresis loops in stress–strain curves. The PLYUNI3 model does not account for stress and strain limits; instead, failure occurs when damage reaches its maximum [66]. Therefore, the damage and plasticity parameters must be determined to capture the stress–strain response accurately.

The parameter identification procedure followed established protocols for CDM models and did not involve automated optimisation algorithms. Instead, parameters were determined through systematic analysis of experimental stress–strain curves from cyclic loading tests. For each loading cycle, elastic and plastic strain components were measured directly from the stress–strain data, and damage variables were calculated from the reduction in reloading modulus compared to the initial undamaged modulus using Equations (10) and (11). Thermodynamic forces were then computed from the measured stress and damage states using Equations (4) and (5). Linear regression (LR) with the least squares method (LSM) was applied to fit the damage evolution laws (Equations (8) and (9)) and the plasticity hardening law (Equation (13)) to the experimental damage-thermodynamic force and stress-plastic strain data, respectively. This direct identification approach based on physical measurements is preferred over inverse optimisation methods. It provides transparent parameter determination with clear physical interpretation and does not require iterative finite element simulations.

**Figure 9:**
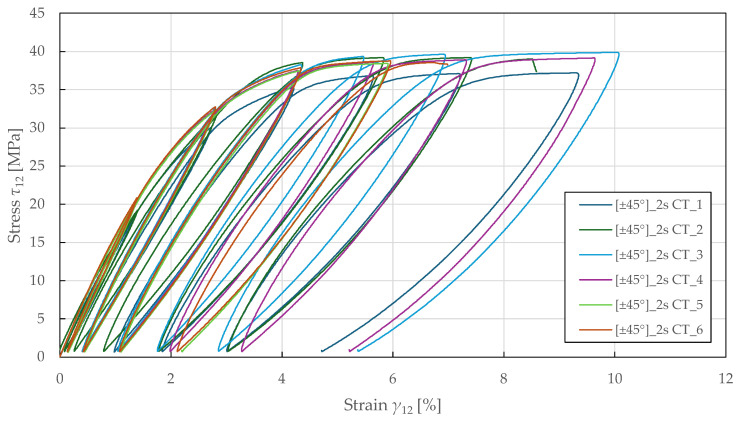
Experimental in-plane shear stress–strain response of FFRPs to CT loading.

The in-plane shear damage and plasticity parameters were determined using ${\left[\pm 45{}^{\circ}\right]}_{2s}$ specimens subjected to cyclic tensile loading. The loading was controlled by extensometer displacement with an increment of 0.6 mm (equivalent to a strain of 0.8%). The resulting stress–strain curves are shown in Figure 9.

Initially, the values of plastic and elastic shear strain and maximum shear stress were analysed from the stress–strain curves to calculate the shear modulus $G_{12i}$ in each cycle i. The undamaged shear modulus was obtained from the initial slope using LR (LSM) in the range of $\tau_{12}=1÷8$ MPa. By comparing the moduli, shear damage was determined from Equation (11):

(20) $$d_{12i}=1-\frac{G_{12i}}{G_{12}^{0}}.$$

This equation quantifies the shear damage variable by comparing the current degraded modulus to the initial undamaged modulus, providing a scalar measure of material stiffness loss ranging from 0 (undamaged) to 1 (complete damage).

Then, assuming the transverse damage is negligible for the specimens with ${\left[\pm 45{}^{\circ}\right]}_{2s}$ lay-up, the damage evolution law was obtained by substituting the shear thermodynamic force from Equation (5) into Equation (7):

(21) $${\underline{Y}}_{i}=\sqrt{Y_{12i}}=\frac{\tau_{12i}}{\sqrt{2G_{12}^{0}}(1-d_{12i})}.$$

The thermodynamic force represents the driving force for damage evolution, analogous to the energy release rate in fracture mechanics. It is calculated from the maximum stress state and the current damage level experienced by the material.

**Figure 10:**
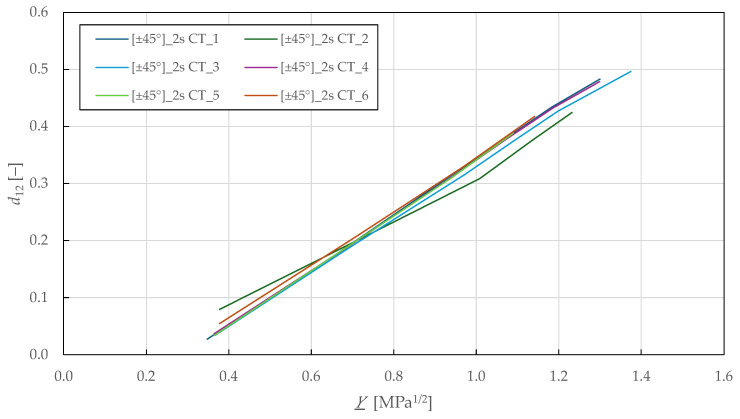
Experimental in-plane shear damage master curves of FFRPs.

Figure 10 shows the $\underline{Y}$-$d_{12}$ shear damage master curves, where a linear relationship is observed up to a shear damage of 0.47 ± 0.03 and thermodynamic force of 1.3 ± 0.04 ${MPa}^{1/2}$. Then, LR (LSM) was used to fit the damage law in Equation (9) to the experimental data, showing a good repeatability of the results. The identified in-plane shear damage parameters for the CDM model are summarised in Table 7.

To obtain the plasticity parameters, the plasticity threshold value was calculated by substituting the effective shear stress from Equation (14) into Equation (12):

(22) $$R_{0}+R_{i}=\frac{\tau_{12i}}{1-d_{12i}}.$$

This expression calculates the current yield stress, considering isotropic hardening, by accounting for the damage effect on the effective stress state. It represents the stress threshold that must be exceeded for continued plastic deformation to occur.

Considering only the in-plane shear strain rate to be non-zero for the specimens with the ${\left[\pm 45{}^{\circ}\right]}_{2s}$ lay-up, the accumulated plastic strain was obtained from Equation (15) as an area under the $\left(1-d_{12}\right)$-$\gamma_{12}^{p}$ curve:

(23) $$p_{i}=\int_{0}^{\gamma_{12i}^{p}}\left(1-d_{12i}\right)d\varepsilon .$$

The accumulated plastic strain is obtained by integrating the plastic strain rate weighted by the damage state. This scalar quantity characterises the total irreversible deformation history and governs the isotropic hardening.

**Figure 11:**
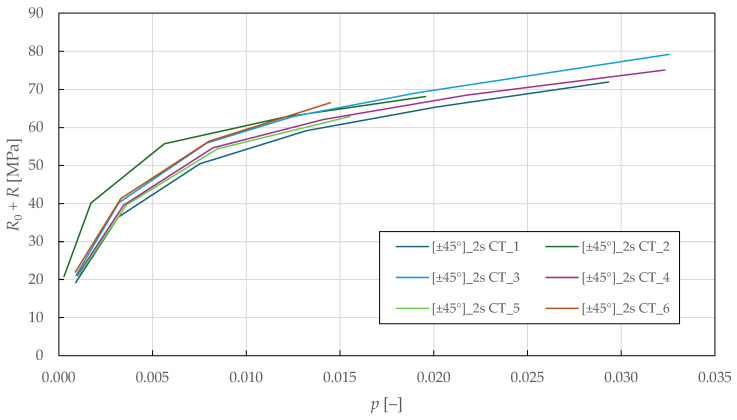
Experimental in-plane shear plasticity master curves of FFRPs.

In Figure 11, the $\left(R_{0}+R\right)$-p shear plasticity master curves are plotted. Then, LR (LSM) was used to fit the isotropic hardening power law in Equation (13) to the experimental data. The identified in-plane shear plasticity parameters for the CDM model are listed in Table 7.

The shear-transverse damage and coupling parameters were identified from the cyclic tensile testing of specimens with ${\left[\pm 22.5{}^{\circ}\right]}_{2s}$ lay-up. The loading was controlled by extensometer displacement with an increment of 0.2 mm (equivalent to a strain of 0.27%). The principal stresses and strains for angle-ply laminates were calculated using classical laminate theory as described in [74]:

(24) $$\sigma_{22i}=\left(1-B\right)\sigma_{xi},$$

(25) $$\tau_{12i}=-\frac{1}{2mn}\left[B\left(1-2m^{2}\right)+m^{2}\right]\sigma_{xi},$$

(26) $$\varepsilon_{22i}=n^{2}\varepsilon_{xi}+m^{2}\varepsilon_{yi},$$

(27) $$\gamma_{12i}=-2mn\left(\varepsilon_{xi}-\varepsilon_{yi}\right),$$

where m=cos22.5°, n=sin22.5°, and B are variables describing the influence of ply angle and undamaged material properties as

(28) $$B=\frac{4m^{2}n^{2}\frac{G_{12}}{E_{2}}\left(\frac{E_{2}}{E_{1}}\nu_{12}+1\right)+m^{2}\left(2m^{2}-1\right)}{4m^{2}n^{2}\frac{G_{12}}{E_{2}}\left(\frac{E_{2}}{E_{1}}+\frac{E_{2}}{E_{1}}\nu_{12}+1\right)+\left(m^{2}-n^{2}\right)\left(2m^{2}-1\right)}.$$

These equations describe the transverse (22) and in-plane (12) stress–strain response of angle-ply laminates as a function of experimentally obtained axial stress (x) and biaxial strain (x,y), ply angle, and material properties in fibre (11), transverse (22), and in-plane (12) directions.

**Figure 12:**
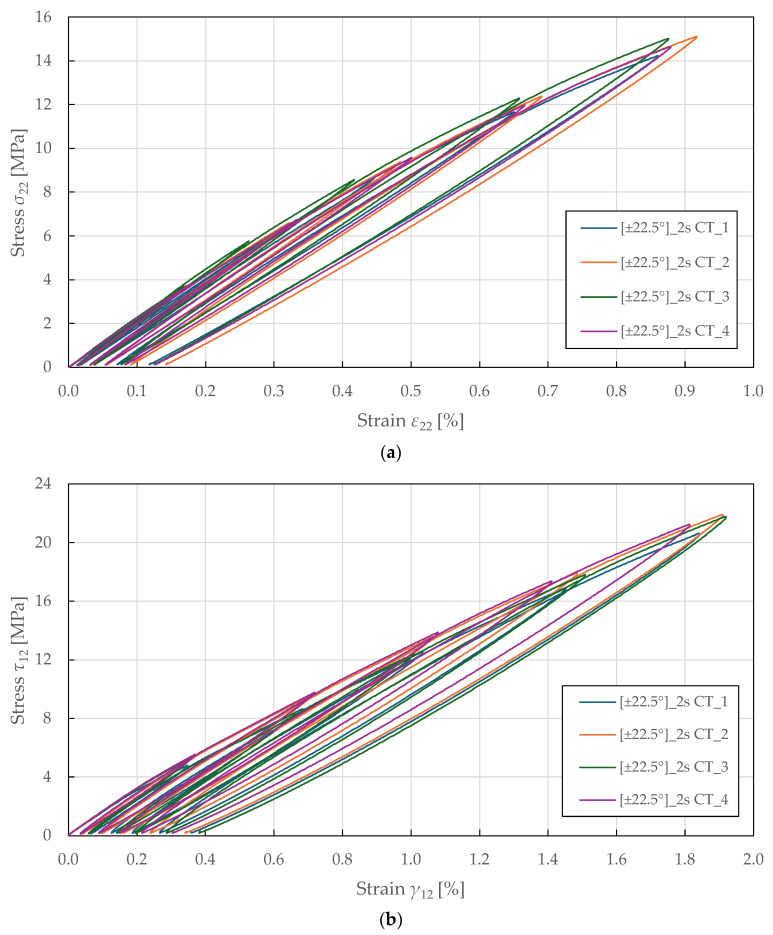
Experimental stress–strain response of ${\left[\pm 22.5{}^{\circ}\right]}_{2s}$ FFRPs to CT loading: (**a**) transverse direction; (**b**) in-plane shear.

The resulting transverse and shear stress–strain curves are plotted in Figure 12. For each i-th cycle, the transverse $E_{2i}$ and shear $G_{12i}$ moduli were determined as described in the previous section. The undamaged moduli were obtained from the initial slopes using LR (LSM) in the absolute stress range of 1 to 2 MPa. From there, the damage variables and thermodynamic forces were determined from Equations (4), (5), (10), and (11).

The shear-transverse damage-coupling parameter $b_{2}$ is described by manipulating Equation (9) and substituting into Equation (7):

(29) $$b_{2}=\frac{{\left(Y_{12\;0}+d_{12i}Y_{12\;c}\right)}^{2}-Y_{12i}}{Y_{22i}},$$

where $Y_{12\;0}$ and $Y_{12\;c}$ are parameters obtained from cyclic tensile testing on the ${\left[\pm 45{}^{\circ}\right]}_{2s}$ specimens (see Table 7). In addition, a coupling parameter $b_{3}$ was introduced to capture the relationship between shear and transverse damage as described in [66]:

(30) $$b_{3}=\frac{d_{22i}}{d_{12i}}.$$

Subsequently, the shear-transverse plasticity coupling parameter a is derived by manipulating the yield conditions in Equation (17) and by substituting the effective stresses and strains from Equations (14) and (15):

(31) $$a^{2}=\frac{\varepsilon_{22i}^{p}{\left(1-d_{22i}\right)}^{2}\tau_{12i}}{\gamma_{12i}^{p}{\left(1-d_{12i}\right)}^{2}\sigma_{22i}}.$$

The physical interpretation of these coupling parameters reflects the complex interaction between failure mechanisms in composite laminates. The parameter b_2_ quantifies how shear damage (fibre–matrix debonding) influences the evolution of transverse damage (matrix microcracking). The parameter b_3_ represents the ratio of transverse to shear damage accumulation rates, characterising the relative progression of these mechanisms. The parameter a governs the coupling between shear-transverse damage and plasticity through effective stress formulation, reflecting the internal friction mechanisms. These parameters are essential for accurately predicting the complex multi-axial behaviour of composite laminates under combined loading, with model sensitivity particularly pronounced for angle-ply laminates.

**Figure 13:**
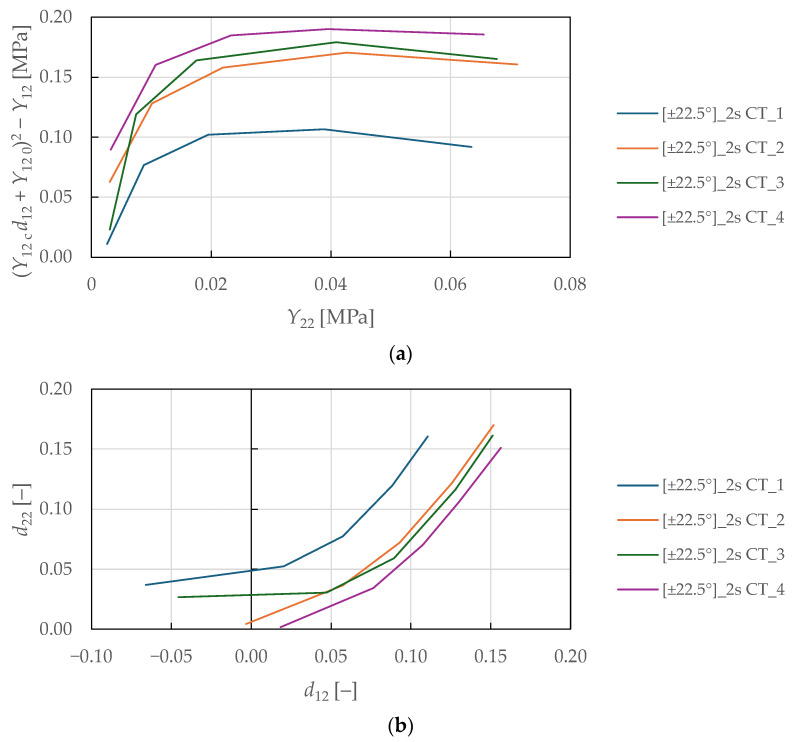

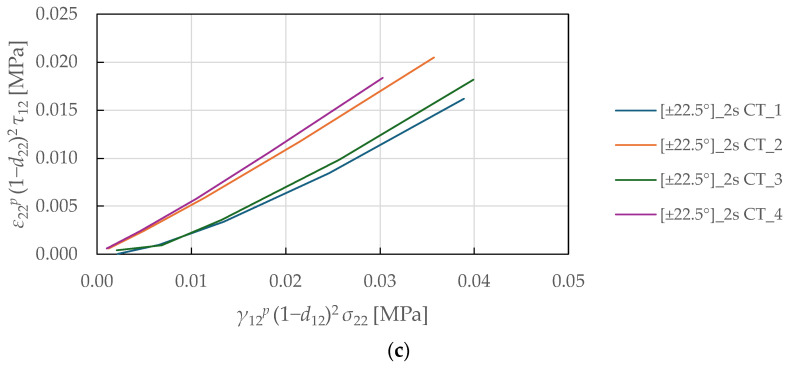
Experimental curves for determination of the shear-transverse coupling parameters of FFRPs: (**a**,**b**) damage-coupling parameters $b_{2}$, $b_{3}$; (**c**) plasticity coupling parameter a.

The coupling parameters represent the slope between the numerator and denominator, which was determined by fitting Equations (29)–(31) to the experimental data plotted in Figure 13a, Figure 13b, and Figure 13c, respectively, using LR (LSM). A nonlinear behaviour was observed in the plotted curves for the shear-transverse damage-coupling parameters $b_{2}$ and $b_{3}$, suggesting that damage coupling in FFRPs intensifies as damage accumulates. This behaviour differs from the linear coupling typically observed in synthetic fibre composites and reflects the complex hierarchical microstructure of natural fibres. Therefore, e.g., a bi-linear evolution law of the parameters would be more suitable for the damage modelling of FFRPs. The identified shear-transverse coupling parameters for the CDM model are listed in Table 8.

**Figure 14:**
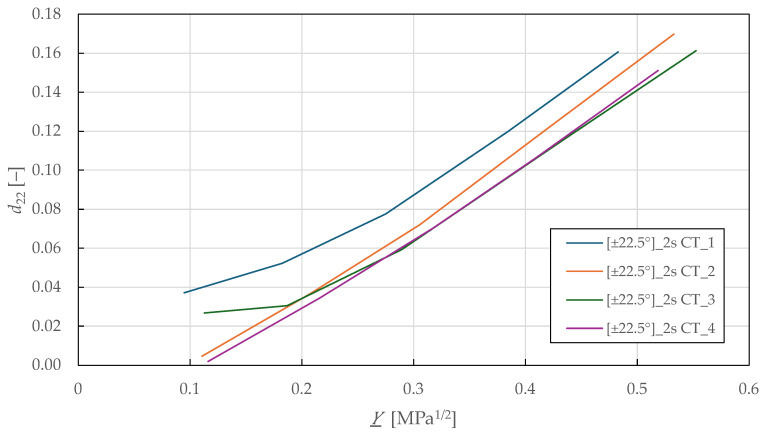
Experimental shear-transverse damage master curves of FFRPs.

Considering the identified coupling parameters and damage evolution law in Equation (7), the $\underline{Y}$-$d_{22}$ shear-transverse damage master curve is plotted in Figure 14. The curves show a mostly linear relationship up to a transverse damage of 0.16 ± 0.01 and thermodynamic force of 0.52 ± 0.03 ${MPa}^{1/2}$. Then, LR (LSM) was used to fit the damage law in Equation (8) to the experimental data. The identified shear-transverse damage parameters for the CDM model are summarised in Table 8.

**Figure 15:**
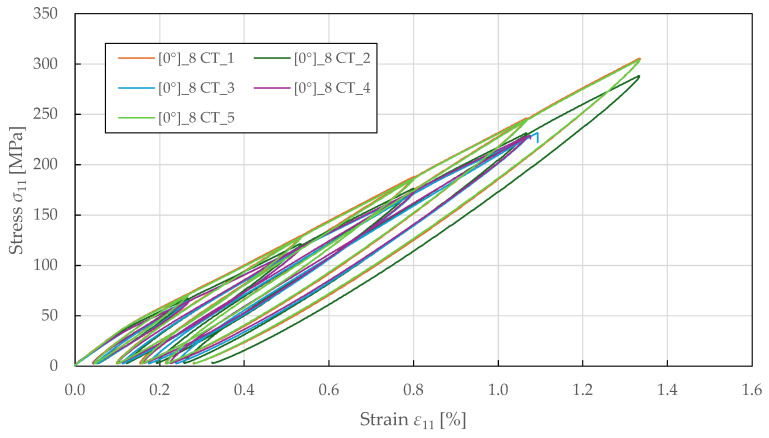
Experimental fibre-direction stress–strain response of FFRPs to CT loading.

The fibre-direction damage parameters were identified from cyclic tensile testing on specimens with ${\left[0\right]}_{8}$ lay-up. The loading was controlled by extensometer displacement with an increment of 0.2 mm (equivalent to a strain of 0.27%). The resulting stress–strain curves are shown in Figure 15. During experimental testing, a progressive loss of stiffness was observed in each loading cycle, indicating a non-brittle failure.

**Figure 16:**
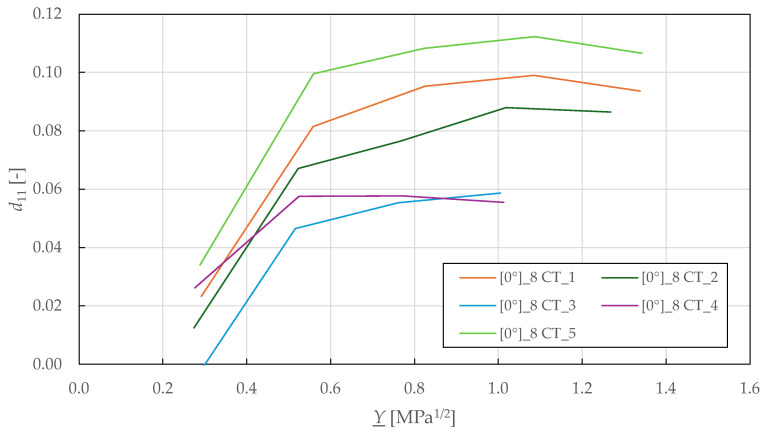
Experimental fibre-direction damage master curves of FFRPs.

Following the procedure described in the previous sections, the $\underline{Y}$-$d_{11}$ fibre-direction damage master curves are plotted in Figure 16. From there, the failure threshold in tension $Y_{11}^{{lim}_{+}\;}$ was determined as the maximum observed thermodynamic force. This value was then adjusted for compression threshold $Y_{11}^{{lim}_{-}\;}$ by considering the difference between the identified tensile and compressive strength (see Table 2 and Table 5). The identified fibre-direction damage parameters for the CDM model are summarised in Table 9.

## 6. Numerical Simulation

To verify and validate the identified material properties of FFRPs, numerical simulations based on the FEM were employed. The FE models were implemented in the Siemens Simcenter Samcef 2412 solver with the intra-laminar CDM mesomodel PLYUNI3. The simulations assumed a quasi-static implicit solution with large deformations. Any imperfections, such as geometric deviations or uneven loading, were not taken into account.

### 6.1. Model Verification on Single-Element Simulation at the Laminate Level

Single-element submodels were used to validate the CDM model. The 1 × 1 × 1 mm linear hexahedral elements were defined as extruded homogenised laminates with lay-ups according to the specimen specification in Table 1. The boundary conditions were defined to represent uniaxial loading.

The intra-laminar behaviour was modelled with mean values of input parameters determined in the previous section. Due to the variability observed across all parameters (average CV of 9.86%), model calibration was necessary to increase the prediction accuracy. The calibrated model parameters are listed in Table 10. The fibre-direction and in-plane shear damage parameters were adjusted within the statistical bounds of ± 2 × SD to align with the experimental stress and strain limits. The parameters for fibre-direction nonlinear stiffness loss, $\xi^{-}$ and $\xi^{+}$, were determined by comparing the results of numerical simulations and experimental testing.

**Figure 17:**
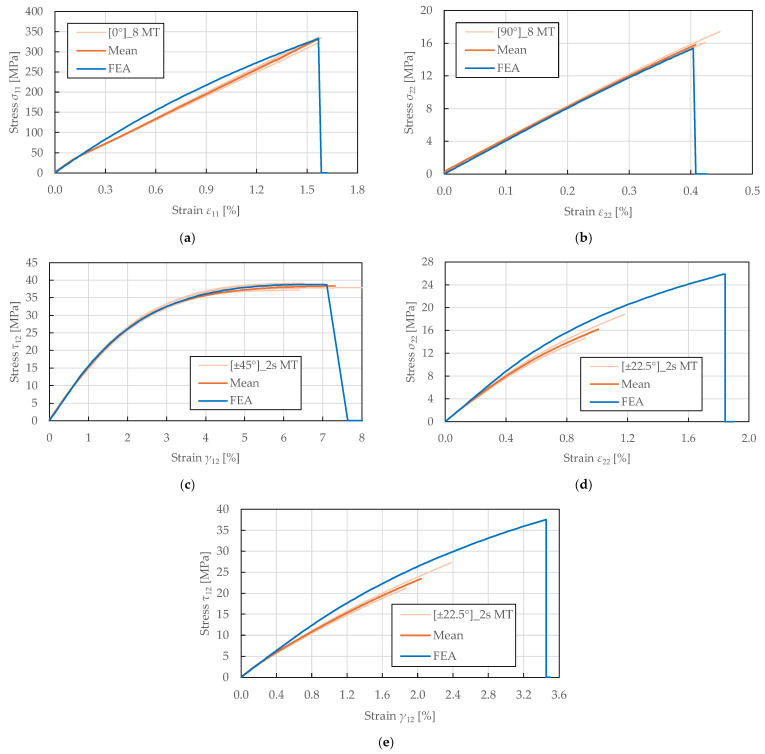
Comparison of stress–strain curves obtained from experimental tensile testing and single-element numerical simulation of FFRPs: (**a**) ${\left[0{}^{\circ}\right]}_{8}$ MT; (**b**) ${\left[90{}^{\circ}\right]}_{8}$ MT; (**c**) ${\left[\pm 45{}^{\circ}\right]}_{2s}$ MT; (**d**,**e**) ${\left[\pm 22.5{}^{\circ}\right]}_{2s}$ MT.

**Figure 18:**
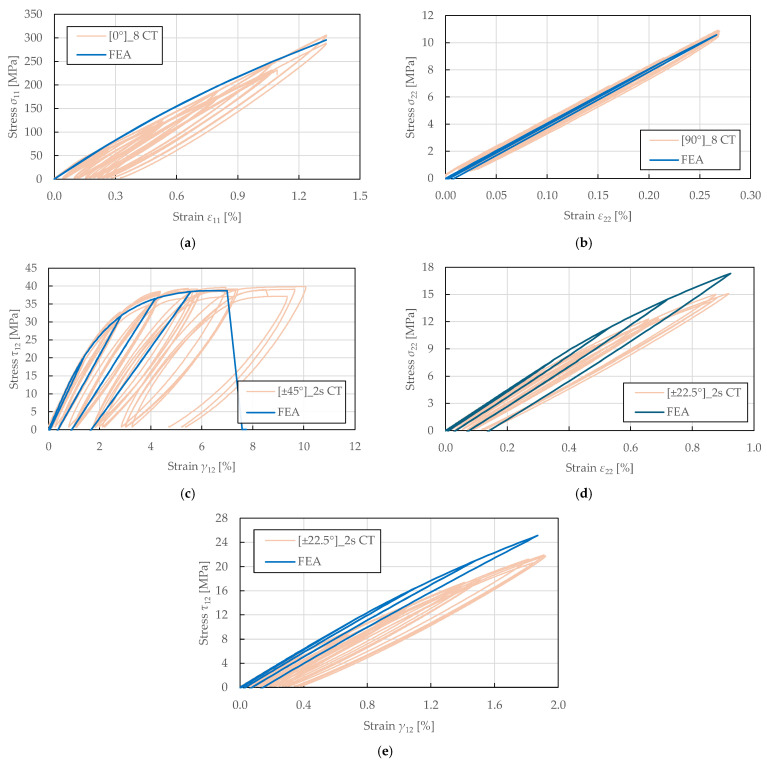
Comparison of stress–strain curves obtained from experimental tensile testing and single-element numerical simulation of FFRPs: (**a**) ${\left[0{}^{\circ}\right]}_{8}$ CT; (**b**) ${\left[90{}^{\circ}\right]}_{8}$ CT; (**c**) ${\left[\pm 45{}^{\circ}\right]}_{2s}$ CT; (**d**,**e**) ${\left[\pm 22.5{}^{\circ}\right]}_{2s}$ CT.

**Figure 19:**
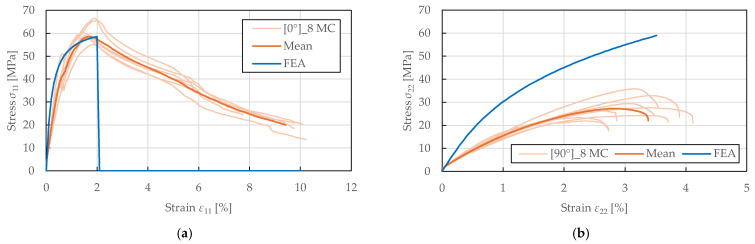
Comparison of stress–strain curves obtained from experimental compressive testing and single-element numerical simulation of FFRPs: (**a**) ${\left[0{}^{\circ}\right]}_{8}$ MC; (**b**) ${\left[90{}^{\circ}\right]}_{8}$ MC.

A comparison of the in-plane behaviour predictions of the calibrated model and the experimental results of FFRPs is presented in Figure 17, Figure 18 and Figure 19, where a good agreement is achieved.

The predicted stress–strain response to monotonic tensile loading is shown in Figure 17. It was found that the initial moduli were accurately predicted for all lay-ups, confirming the correctness of the determined elastic properties. Additionally, the model captured the stress–strain curves to their full extent for the transverse direction and in-plane shear, as plotted in Figure 17b,c. The predicted in-plane shear response accuracy confirmed the correctness of the identified plasticity parameters. In the fibre-direction, the modulus past the yield stress was slightly overpredicted, as presented in Figure 17a. These findings confirm the suitability of the PLYUNI3 model for damage modelling of FFRPs for the 0°, 90°, and ±45° configurations. The simplified damage and plasticity formulation in the fibre and transverse directions provides sufficient accuracy for structural engineering applications.

In the principal directions, a close match to the identified tensile strengths and ultimate strains was achieved (differences of 0.73%, 2.76%, 0.63% to stress limits, and 0.51%, 1.46%, 2.58% to strain limits), as depicted in Figure 17a–c. However, the fibre-dominated failure was largely overpredicted for the transverse and shear responses of ${\left[\pm 22.5\right]}_{2s}$ lay-up (differences of 60% to stress limits and 82.48%, 69.35% to strain limits), as shown in Figure 17d,e. Despite this, the prediction roughly corresponded to the experimental results below the ultimate strains of approximately 1% and 2%. The insufficient progression of shear and transverse damage can be attributed to the linear shear-transverse coupling formulation.

The predicted stress–strain response to cyclic tensile loading is shown in Figure 18. It was observed that the hysteresis loops were not fully captured. Instead, the model predicts the damaged moduli and plastic strains exhibited upon reloading, which corresponds to the PLYUNI3 model formulation that does not account for unloading–reloading nonlinearity. The linear reloading paths reflect the assumption of constant damage during unloading, while the reduced stiffness and permanent plastic strains accurately represent the damage and plasticity accumulated during prior loading.

For the fibre-direction response in Figure 18a, the predicted stress–strain curve exhibited no plasticity upon reloading despite the observed nonlinearity. This is due to the definition of plasticity development law, which only accounts for the accumulation of shear damage. The damage evolution in the fibre direction is characterised by a stiffness loss parameter up to a sudden failure at the damage threshold. For the transverse direction in Figure 18b, the magnitude of damage was underpredicted in the initial cycles, though the overall trend matched the experimental behaviour. In contrast, an excellent agreement was achieved for the in-plane shear response plotted in Figure 18c, accurately matching the shear damage and plasticity master curves. The hysteresis loop shapes, reloading moduli, and permanent strains were well-predicted, confirming the correctness of the identified shear damage and plasticity parameters. For the ${\left[\pm 22.5{}^{\circ}\right]}_{2s}$ lay-up, the damaged moduli were overpredicted in the transverse and in-plane responses depicted in Figure 18d,e. This can be attributed to the simplified linear coupling assumptions that do not fully capture the nonlinear damage interaction observed experimentally in this configuration.

The predicted stress–strain response to monotonic compressive loading is shown in Figure 19. For the fibre-direction response presented in Figure 19a, a good prediction accuracy was achieved up to compressive strength (difference of 3.52%). The model successfully captured the initial nonlinearity and yield point. However, the post-peak softening behaviour could not be predicted due to stability issues (buckling and bending of specimens) that are not accounted for in the material model. In contrast, the failure in the transverse direction was not captured, and the stress response was significantly overpredicted, as presented in Figure 19b. This limitation arises from the model’s assumption that matrix microcracks close under compression, preventing the evolution of transverse damage. Consequently, only plasticity develops in transverse compression without the stiffness degradation observed experimentally. This represents a known limitation of the Ladevèze–LeDantec model formulation for materials exhibiting progressive crushing damage in transverse compression, which is particularly pronounced in FFRPs due to their cellular structure and lower compressive strength.

### 6.2. Model Validation on Open-Hole Tensile Test

**Figure 20:**
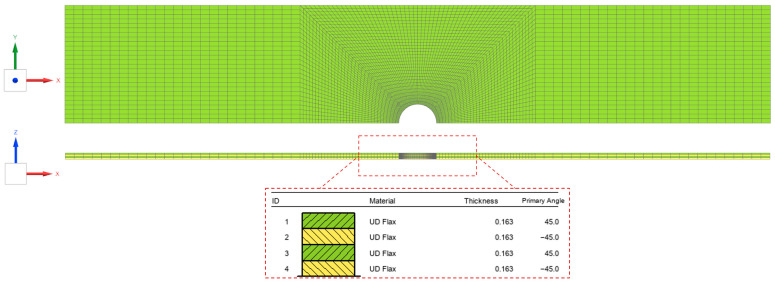
FE model of FFRP specimens in OHT configuration.

The OHT specimens were used to validate the calibrated CDM model. The dimensions were defined according to Figure 1b and Table 1. Due to symmetry in the longitudinal-transverse plane, only 1/2 of the gauge section was modelled to increase computational efficiency. Linear hexahedral elements were used for discretisation, and local mesh refinement was defined around the centre hole. The composite FE models were extruded with one element per ply, as shown in Figure 20.

To achieve better convergence of the nonlinear solution, a time delay function was enabled with parameters $a_{c}=1$ and $\tau_{c}=0.02\;s$, which was approximately equal to 25/1000 of the analysis time. The boundary conditions were defined as uniaxial loading.

**Figure 21:**
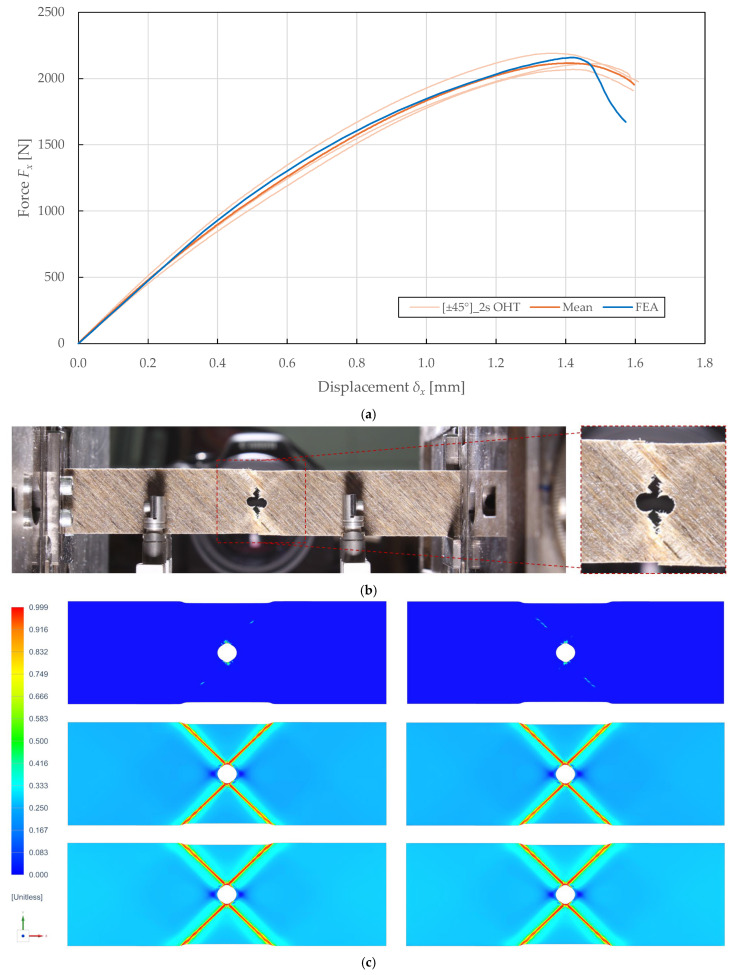
Comparison of results obtained from experimental OHT testing and numerical simulation of FFRPs with ${\left[\pm 45{}^{\circ}\right]}_{2s}$ lay-up: (**a**) force-displacement curves; (**b**) observed failure; (**c**) results of predicted damage $d_{11}\;[-]$, $d_{22}\;[-]$, $d_{12}\;[-]$ (top to bottom) for the 0° plies.

**Figure 22:**
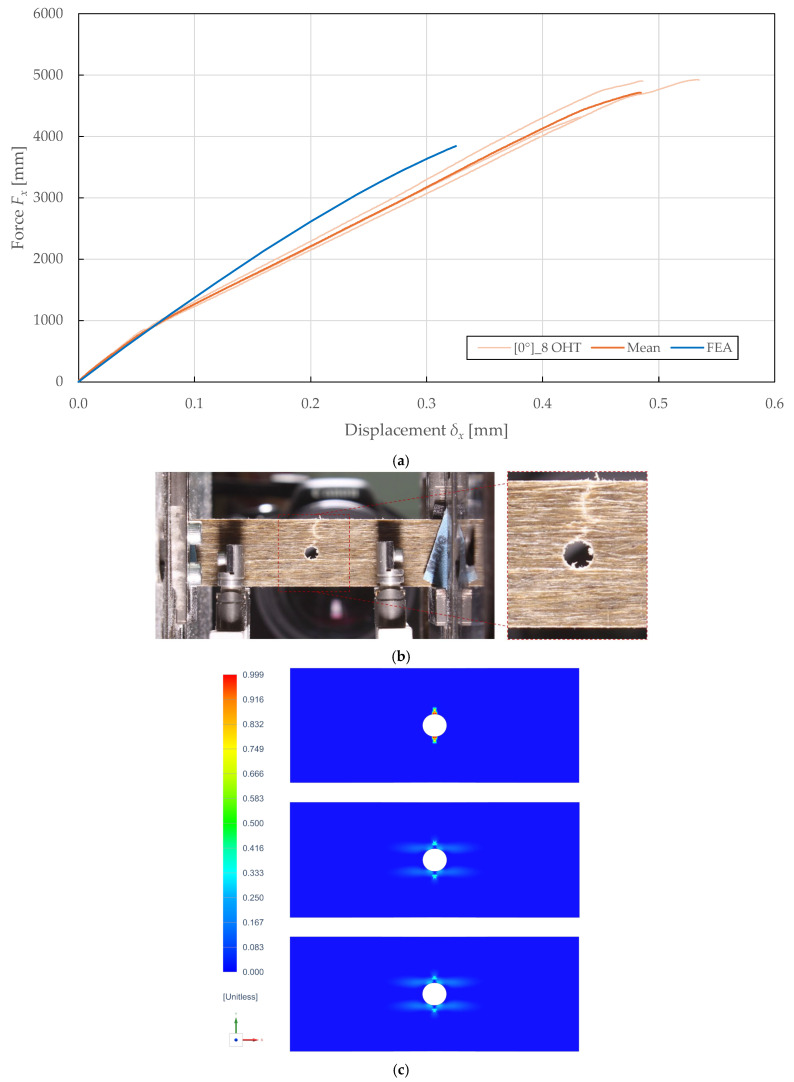
Comparison of results obtained from experimental OHT testing and numerical simulation of FFRPs with ${\left[0{}^{\circ}\right]}_{8}$ lay-up: (**a**) force-displacement curves; (**b**) observed failure; (**c**) results of predicted damage $d_{11}\;[-]$, $d_{22}\;[-]$, $d_{12}\;[-]$ (top to bottom) for the ± 45° adjacent plies (left to right).

A comparison of the in-plane behaviour predictions of the calibrated model and the experimental results of OHT specimens is presented in Figure 21 and Figure 22. Experimental testing on the ${\left[0{}^{\circ}\right]}_{8}$ and ${\left[\pm 45{}^{\circ}\right]}_{2s}$ lay-ups according to ASTM D5766 [72] revealed maximum forces of 2132.35 ± 122.95 N (CV of 2.90%) and 4714.30 ± 694.62 N (CV of 7.37%) at displacements of 1.42 ± 0.12 mm (CV of 4.19%) and 0.48 ± 0.10 mm (CV of 10.35%), respectively.

The failure modes were consistent across the specimens and are shown in Figure 21b and Figure 22b. In particular, an alternation between fibre–matrix debonding and fibre rupture of the adjacent ± 45° plies, and a combination of fibre rupture and matrix microcracking of the 0° plies was observed.

The calibrated model predicted the force-displacement curves with an accuracy of 98.85% and 67.59%, as plotted in Figure 21a and Figure 22a. The prediction accuracy was quantified by considering the ratios of the predicted maximum force, its corresponding displacement, and deformation energy (i.e., the area under the force-displacement curve) to the experimental mean results. The underprediction of maximum force for the ${\left[0{}^{\circ}\right]}_{8}$ was attributed to convergence issues associated with the sudden failure.

The predicted damage distributions presented in Figure 21c and Figure 22c correlate well with the experimentally observed failure modes. For the ${\left[\pm 45{}^{\circ}\right]}_{2s}$ specimen, the model predicted localised fibre-direction damage ($d_{11}$) at the hole edge in regions corresponding to the observed fibre rupture, while elevated shear damage ($d_{12}$) extended along the net-section width, consistent with the observed fibre–matrix debonding patterns. The alternating damage patterns between adjacent ±45° plies correspond to the experimental observation of alternating fibre–matrix debonding and fibre rupture between plies. For the ${\left[0{}^{\circ}\right]}_{8}$ specimen, the model predicted severe transverse damage ($d_{22}$) in narrow bands perpendicular to the loading direction emanating from the hole edge, corresponding to the matrix microcracking observed experimentally. The localised fibre-direction damage ($d_{11}$) at the minimum net-section corresponds to the final fibre rupture failure mode. The shear damage ($d_{12}$) remained relatively low throughout the specimen, consistent with the predominantly tension-dominated failure mode. These correlations demonstrate that the model correctly captures the spatial distribution and progression sequence of different damage mechanisms, validating its applicability for damage-tolerant design and virtual prototyping of notched FFRP structures.

### 6.3. Model Prediction Capabilities for Virtual Prototyping

The calibrated CDM model was verified and validated in the previous sections. Considering the increasing use of FEA for virtual prototyping, the model prediction capabilities were explored on the OHT specimens. In particular, the effects of discretisation, damage localisation, and linear prediction were studied, with details summarised in Table 11. In conclusion, the FEA setup #8 was found to have a superior cost–accuracy ratio, making it the most suitable for fast design assessment in engineering applications.

**Figure 23:**
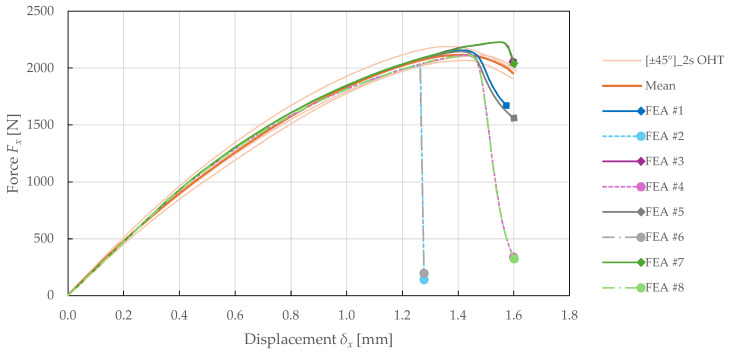
Force-displacement curves obtained from experimental ${\left[\pm 45\right]}_{2s}$ OHT and numerical simulations of FFRPs with different FEA setups.

**Figure 24:**
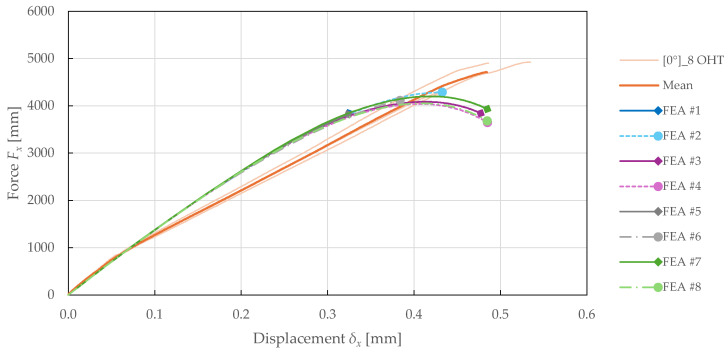
Force-displacement curves obtained from experimental ${\left[0{}^{\circ}\right]}_{8}$ OHT and numerical simulations of FFRPs with different FEA setups.

The comparison of predicted force-displacement curves for each FEA setup is presented in Figure 23 and Figure 24. The results suggest that the model is independent of the number of elements in the 3rd direction. This can be attributed to the use of a purely in-plane behaviour formulation of the PLYUNI3 model. The FEA with a homogenised laminate also showed a significant increase in computational efficiency.

When using the non-local approach for damage calculation, a delay in failure was observed. This resulted in better simulation convergence for the ${\left[0{}^{\circ}\right]}_{8}$ specimen, and an overprediction of strength for the ${\left[\pm 45{}^{\circ}\right]}_{2s}$ specimen. On the contrary, FEA solved with linear prediction exhibited premature failure. However, using both NLC and LP allowed for accurate failure prediction.

## 7. Conclusions

In this study, an advanced intra-laminar CDM mesomodel PLYUNI3 was validated for FFRP laminates manufactured from FLAXPREG T-UD 100 prepregs. Extensive quasi-static experimental testing (MT, CT, MC, OHT) was performed on a Zwick/Roell Z050 electromechanical testing machine to study the stress–strain response and damage behaviour on multiple specimen configurations (0°, 90°, ±45°, and ±22.5°). The empirical data showed good repeatability across all specimens. A full suite of elastic, strength, and damage parameters was identified with an average CV of 9.86%. The observed variability is comparable to that reported in the literature for similar natural fibre materials tested under controlled conditions. It arises from natural fibre heterogeneity and manufacturing variations rather than purely statistical sampling effects.

The model was verified and calibrated on the single-element simulations and validated through OHT in Siemens Simcenter Samcef 2412. For engineering design applications, the calibrated model parameters represent mean material behaviour suitable for deterministic analysis with appropriate safety factors. This approach is consistent with current industry practice for composite structural design. The comparison of predicted and experimental results in OHT validation tests on specimens with ±45° and 0° lay-up configurations showed excellent agreement, achieving overall force-displacement prediction accuracies of 97.61% and 88.98%, respectively. In particular, the differences from empirical results, i.e., maximum force, displacement, and deformation energy, were 0.40%, 1.33%, 1.50%, and 14.03%, 15.35%, 8.56% for ±45° and 0° configurations. The model fully captured the failure modes and matched the damage distribution. These results confirm the relevance of the Ladevèze–LeDantec framework and demonstrate the model’s capabilities for accurate damage assessment in FFRP design.

Several significant limitations of the CDM approach for FFRPs were identified in this study, reflecting specific formulation choices rather than fundamental incompatibility with natural fibres. Most significantly, the PLYUNI3 model failed to predict transverse compressive failure, significantly overpredicting the stress response. This limitation stems from a fundamental assumption in the Ladevèze–LeDantec model that matrix microcracks close under compression, and the transverse damage evolution law depends solely on positive (tensile) transverse stress. Consequently, the model predicts only plastic deformation without the progressive crushing and kinking damage mechanisms that dominate NFC behaviour in transverse compression. This is particularly problematic for FFRPs due to their cellular microstructure and lower compressive strength compared to synthetic fibre composites. Addressing this limitation would require modification of the damage evolution law to include a compressive damage mechanism, which is beyond the scope of the present work but represents an important direction for future model development.

Furthermore, the underprediction of failure loads for specific angle-ply configurations suggests that the linear damage-coupling assumptions may be oversimplified for NFCs, which exhibit more complex damage interaction mechanisms. Furthermore, due to the lack of coupling to fibre damage and the current formulation of damage and plasticity laws, the model is most effective in predicting the behaviour of laminates with shear-dominated lay-ups.

Finally, the model requires numerical stabilisation techniques to achieve robust convergence for complex structural configurations, particularly those involving sudden fibre-dominated failure modes. While these requirements increase computational complexity compared to simple failure criteria, they are typical for advanced progressive damage analysis and are readily implemented in modern commercial FE codes. For FFRPs, this limitation was resolved by introducing a time delay equivalent to 25/1000 of the analysis time, and the use of the NLC approach with the LP solution. This FEA setup exhibited a significant increase in efficiency while maintaining accuracy, as demonstrated through validation on OHT specimens.

An important consideration for practical application concerns the influence of parameter variability on model predictions. The observed CV for identified parameters ranged from 1.92% to 27.67%, averaging 9.86%. The highest specimen-to-specimen scatter was identified for coupling parameters ($b_{2}$, $b_{3}$) and transverse damage initiation threshold ($Y_{22\;0}$). While formal stochastic analysis was beyond the scope of this initial validation study, the successful prediction of validation test results using mean parameter values demonstrates adequate robustness for deterministic engineering analysis with appropriate safety factors. The parameter variability primarily affects the precise timing of damage initiation and the slope of damage evolution curves rather than the overall failure modes and ultimate strength predictions. Parameters governing elastic properties and ultimate failure thresholds exhibited lower variability (CV < 10%), providing confidence in strength predictions. For applications requiring probabilistic design approaches, the characterised parameter distributions provide input for Monte Carlo or stochastic finite element analysis. Future work should include formal sensitivity analysis to identify which parameters most strongly influence key design metrics and to establish guidelines for parameter variability acceptable for different application requirements. Comparison of the experimental validation results (prediction accuracies of 97.61% and 88.98% for force-displacement response in OHT) against the average CV of 9.86% suggests that the model framework effectively integrates the distributed parameter uncertainty into useful engineering predictions. The practical value lies not in achieving perfect parameter fidelity but in providing validated quantitative predictions that are superior to simplified approaches while accounting for material variability through established safety factor methodologies.

Another important consideration when applying CDM models developed for synthetic fibres to NFC concerns whether the unique micromechanical characteristics of natural fibres—including hierarchical cellular structure, lumen presence, and variable fibre–matrix adhesion—necessitate modifications to the damage evolution laws. The original Ladevèze–LeDantec model formulation was applied in the present study without modifying the fundamental damage evolution laws or coupling definitions. The natural fibre characteristics are implicitly accommodated through the calibrated model parameters rather than by reformulating the evolution laws. The identified damage thresholds, critical values, and coupling parameters reflect the effective mesoscale behaviour of the flax–epoxy composite, including the influence of fibre microstructure. This approach is consistent with the mesoscale philosophy of CDM, which treats the ply as a homogenised continuum without explicitly modelling micro-scale features. The successful validation demonstrated that this level of modelling is sufficient for engineering design applications. However, the observed nonlinear evolution of coupling parameters suggests that enhanced damage evolution laws incorporating nonlinear coupling might improve prediction accuracy for complex loading scenarios. Future work could explore whether incorporating fibre microstructure effects through modified evolution laws or stochastic parameter distributions would improve predictions for a broader range of natural fibre systems.

This paper extends the CDM theory to NFCs. Integrating experimental material characterisation with FEA provides a robust framework for virtual prototyping of sustainable composite structures, enabling design optimisation and damage tolerance assessment while reducing the number of expensive structural-level validation tests, accelerating time-to-market. These findings establish a foundation for future research on optimisation strategies, environmental effect modelling, and lifecycle prediction, which are essential for the broader adoption of sustainable composites in structural applications. In the automotive sector, the methodology supports virtual prototyping of interior panels, seat structures, and non-safety-critical components where FFRPs can replace synthetic composites while reducing weight and environmental impact. For marine applications, the damage-tolerant design capability is essential for hull panels and deck structures subjected to complex multi-axial loading. In construction, the approach enables design optimisation of façade elements, interior partition systems, and temporary structures where FFRP offers advantages in terms of carbon footprint reduction and end-of-life recyclability. The identified limitations define priority areas for future model enhancement. Future research could expand the calibrated model to include delamination and interlaminar damage by coupling with interface models. Furthermore, a multi-scale approach could be derived to reduce parameter uncertainty arising from natural fibre variability. Finally, the validated model can be leveraged to design optimisation of eco-friendly structures in the automotive, marine, and construction sectors to accelerate NFC adoption.

## Figures and Tables

**Table 1:** Experimental test matrix for characterising material properties for damage modelling of FFRPs (all dimensions in mm).

Lay-Up	l	w	t	g (Tabs)	∅d	Test (Sample Size) ^1^	Instrumentation ^2^	Test Output
${\left[0{}^{\circ}\right]}_{8}$	250	15	1.3	50 (no)	-	MT (4), CT (5)	AE *, BE	$E_{1},\;\nu_{12},\;X^{T},\;\varepsilon_{11}^{T},\;Y_{11}^{{lim}_{+}},\;\xi^{+}$
${\left[90{}^{\circ}\right]}_{8}$	175	25	1.3	30 (no)	-	MT (4), CT (4)	AE *	$E_{2},\;Y^{T},\;\varepsilon_{22}^{T}$
${\left[\pm 45\right]}_{2s}$	250	25	1.3	50 (no)	-	MT (5), CT (6)	AE *, BE	$G_{12},\;S_{12},\;Y_{12\;0},\;Y_{12\;c},\;Y_{12\;s},\;R_{0},\;R\left(p\right)$
${\left[\pm 22.5\right]}_{2s}$	250	25	1.3	50 (no)	-	MT (4), CT (4)	AE *, BE	$Y_{22\;0},\;Y_{22\;c},\;Y_{22\;s},\;b_{2},\;b_{3},a$
${\left[0{}^{\circ}\right]}_{8}$	150	10	1.3	65 (yes)	-	MC (6)	-	$X^{C},\;\varepsilon_{11}^{C},\;Y_{11}^{{lim}_{-}},\;\xi^{-}$
${\left[90{}^{\circ}\right]}_{8}$	150	10	1.3	65 (yes)	-	MC (8)	-	$Y^{C},\varepsilon_{11}^{C}$
${\left[\pm 45\right]}_{2s}$	250	25	1.3	50 (no)	4	OHT (3)	AE *	validation
${\left[0{}^{\circ}\right]}_{8}$	150	20	1.3	40 (no)	4	OHT (3)	AE **	validation

^1^ MT, monotonous tensile; CT, cyclic tensile; MC, monotonous compressive; OHT, open-hole tensile. ^2^ AE, automatic axial extensometer (*—GL 75 mm; **—GL 50 mm); BE, clip-on biaxial extensometer.

**Table 2:** Identified tensile fibre-direction material properties of FFRPs.

Property	Obtained From	Method ^1^	Evaluation Range	Mean ± 2 × SD (CV)	Unit
$E_{1}$	${\left[0{}^{\circ}\right]}_{8}$ MT	LR (LSM)	$\sigma_{11}=5÷30\;MPa$	29.88 ± 2.71 (4.54%)	GPa
$\nu_{12}$	${\left[0{}^{\circ}\right]}_{8}$ MT	LR (LSM)	$\varepsilon_{11}=0.1÷0.3\%$	0.48 ± 0.05 (5.23%)	-
$X^{T}$	${\left[0{}^{\circ}\right]}_{8}$ MT	-	-	330.07 ± 13.65 (2.07%)	MPa
$\varepsilon_{11}^{T}$	${\left[0{}^{\circ}\right]}_{8}$ MT	-	-	1.56 ± 0.06 (1.92%)	%

^1^ LR (LSM), linear regression (least squares method).

**Table 3:** Identified tensile transverse direction material properties of FFRPs.

Property	Obtained From	Method ^1^	Evaluation Range	Mean ± 2 × SD (CV)	Unit
$E_{2}$	${\left[90{}^{\circ}\right]}_{8}$ MT	LR (LSM)	$\sigma_{22}=1÷2\;MPa$	4.08 ± 0.33 (4.10%)	GPa
$Y^{T}$	${\left[90{}^{\circ}\right]}_{8}$ MT	-	-	15.81 ± 2.83 (8.94%)	MPa
$\varepsilon_{22}^{T}$	${\left[90{}^{\circ}\right]}_{8}$ MT	-	-	0.41 ± 0.08 (9.61%)	%

^1^ LR (LSM), linear regression (least squares method).

**Table 4:** Identified in-plane shear material properties of FFRPs.

Property	Obtained From	Method ^1^	Evaluation Range	Mean ± 2 × SD (CV)	Unit
$G_{12}$	${\left[\pm 45{}^{\circ}\right]}_{2s}$ MT	LR (LSM)	$\tau_{12}=1÷8\;MPa$	1.62 ± 0.12 (3.72%)	GPa
$S_{12}$	${\left[\pm 45{}^{\circ}\right]}_{2s}$ MT	-	-	38.49 ± 1.70 (2.21%)	MPa
$\gamma_{12}$	${\left[\pm 45{}^{\circ}\right]}_{2s}$ MT	-	-	7.29 ± 3.85 (26.39%)	%

^1^ LR (LSM), linear regression (least squares method).

**Table 5:** Identified compressive fibre-direction material properties of FFRPs.

Property	Obtained From	Mean ± 2 × SD (CV)	Unit
$X^{C}$	${\left[0{}^{\circ}\right]}_{8}$ MC	60.64 ± 8.93 (7.36%)	MPa
$\varepsilon_{11}^{C}$	${\left[0{}^{\circ}\right]}_{8}$ MC	1.74 ± 0.41 (11.91%)	%

**Table 6:** Identified compressive transverse direction material properties of FFRPs.

Property	Obtained From	Mean ± 2 × SD (CV)	Unit
$Y^{C}$	${\left[90{}^{\circ}\right]}_{8}$ MC	27.60 ± 9.64 (17.47%)	MPa
$\varepsilon_{22}^{C}$	${\left[90{}^{\circ}\right]}_{8}$ MC	2.97 ± 0.96 (16.18%)	%

**Table 7:** Identified in-plane shear damage and plasticity parameters of FFRPs.

Parameter	Obtained From	Method ^1^	Evaluation Range	Mean ± 2 × SD (CV)	Unit
$Y_{12\;0}$	${\left[\pm 45{}^{\circ}\right]}_{2s}$ CT	LR (LSM)	full	0.28 ± 0.07 (13.10%)	${MPa}^{1/2}$
$Y_{12\;c}$	${\left[\pm 45{}^{\circ}\right]}_{2s}$ CT	LR (LSM)	full	2.17 ± 0.31 (7.13%)	${MPa}^{1/2}$
$Y_{12\;s}$	${\left[\pm 45{}^{\circ}\right]}_{2s}$ CT	LR (LSM)	full	1.27 ± 0.15 (6.09%)	${MPa}^{1/2}$
$R_{0}$	${\left[\pm 45{}^{\circ}\right]}_{2s}$ CT	-	-	22.21 ± 2.06 (4.63%)	MPa
K	${\left[\pm 45{}^{\circ}\right]}_{2s}$ CT	LR (LSM)	full	294.45 ± 112.44 (19.09%)	MPa
γ	${\left[\pm 45{}^{\circ}\right]}_{2s}$ CT	LR (LSM)	full	0.36 ± 0.09 (12.68%)	-

^1^ LR (LSM), linear regression (least squares method).

**Table 8:** Identified shear-transverse damage and coupling parameters of FFRPs.

Parameter	Obtained From	Method ^1^	Evaluation Range	Mean ± 2 × SD (CV)	Unit
$Y_{22\;0}$	${\left[\pm 22.5{}^{\circ}\right]}_{2s}$ CT	LR (LSM)	full	0.10 ± 0.05 (26.61%)	${MPa}^{1/2}$
$Y_{22\;c}$	${\left[\pm 22.5{}^{\circ}\right]}_{2s}$ CT	LR (LSM)	full	2.86 ± 0.58 (10.18%)	${MPa}^{1/2}$
$Y_{22\;s}$	${\left[\pm 22.5{}^{\circ}\right]}_{2s}$ CT	LR (LSM)	full	0.54 ± 0.06 (5.38%)	${MPa}^{1/2}$
$b_{2}$	${\left[\pm 22.5{}^{\circ}\right]}_{2s}$ CT	LR (LSM)	full	1.26 ± 0.53 (22.14%)	-
$b_{3}$	${\left[\pm 22.5{}^{\circ}\right]}_{2s}$ CT	LR (LSM)	full	0.86 ± 0.47 (27.67%)	-
a	${\left[\pm 22.5{}^{\circ}\right]}_{2s}$ CT	LR (LSM)	full	0.73 ± 0.11 (7.32%)	-

^1^ LR (LSM), linear regression (least squares method).

**Table 9:** Identified fibre-direction damage parameters of FFRPs.

Property	Obtained From	Mean ± 2 × SD (CV)	Unit
$Y_{11}^{{lim}_{+}\;}$	${\left[0{}^{\circ}\right]}_{8}$ CT	1.23 ± 0.35 (14.26%)	${MPa}^{1/2}$
$Y_{11}^{{lim}_{-}\;}$	${\left[0{}^{\circ}\right]}_{8}$ CT	0.23 ± 0.07 (16.18%)	${MPa}^{1/2}$

**Table 10:** Calibrated CDM model input parameters of FFRPs.

Parameter	Value	Unit	Adjustment
$Y_{11}^{{lim}_{+}\;}$	1.35	${MPa}^{1/2}$	5.76%
$Y_{11}^{{lim}_{-}\;}$	0.24	${MPa}^{1/2}$	6.32%
$Y_{12\;0}$	0.24	${MPa}^{1/2}$	−14.24%
$Y_{12\;c}$	2.38	${MPa}^{1/2}$	9.54%
$\xi^{+}$	−28	-	-
$\xi^{-}$	450	-	-

**Table 11:** FEA setup matrix for determining the CDM model prediction capabilities.

FEA Setup	Discretisation ^1^	Damage ^2^	Linear Prediction	Computational Cost	Prediction Accuracy ^3^
#1	full-scaled	local	no	-	98.85%/67.59%
#2	full-scaled	local	yes	−75.68%	84.67%/89.87%
#3	full-scaled	NLC (0.1625)	no	−12.75%	94.62%/89.97%
#4	full-scaled	NLC (0.1625)	yes	−12.16%	97.94%/88.74%
#5	homogenised	local	no	−32.67%	99.25%/67.17%
#6	homogenised	local	yes	−82.64%	84.67%/79.54%
#7	homogenised	NLC (0.1625)	no	−28.86%	94.53%/89.81%
#8	homogenised	NLC (0.1625)	yes	−42.78%	97.61%/88.98%

^1^ Full-scaled (one element per elementary ply); homogenised (one element per laminate). ^2^ NLC, non-local continuous approach (diffusion radius in mm). ^3^ Results for OHT with ${\left[\pm 45\right]}_{2s}$/${\left[0{}^{\circ}\right]}_{8}$ lay-ups.

## Data Availability

The original contributions presented in the study are included in the article; further inquiries can be directed to the corresponding author.

## Abbreviations

The following abbreviations are used in this manuscript:

AE	Axial Extensometer
BE	Biaxial Extensometer
CDM	Continuum Damage Mechanics
CFRP	Carbon Fibre-Reinforced Polymer
CS	Coordinate System
CT	Cyclic Tensile
CV	Coefficient of Variation
FE	Finite Element
FFRP	Flax Fibre-Reinforced Polymer
GFRP	Glass Fibre-Reinforced Polymer
GL	Gauge Length
LCA	Life Cycle Assessment
LP	Linear Prediction
LR (LSM)	Linear Regression (Least Squares Method)
MC	Monotonous Compressive
MT	Monotonous Tensile
NFC	Natural Fibre Composite
NLC	Non-Local Continuous
OHT	Open-Hole Tensile
SD	Standard Deviation
UD	Unidirectional
WF	Woven Fabric
